# Stable Synapse‐Like Memory Switching in N‐Heterocyclic Carbene Monolayers

**DOI:** 10.1002/anie.1823213

**Published:** 2026-04-17

**Authors:** Ankita Das, Alessandro Borrini, Christian Gutheil, Björn Braunschweig, Billura Shakhayeva, Georgios Katsoukis, Ab F. Nieuwenhuis, Raka Ahmed, Susanne Leitherer, Gemma C. Solomon, Frank Glorius, Christian A. Nijhuis

**Affiliations:** ^1^ Organisch‐Chemisches Institut University of Münster Münster Germany; ^2^ Hybrid Materials for Opto‐Electronics Group Department of Molecules and Materials MESA+ Institute for Nanotechnology Molecules Center and Center for Brain‐Inspired Nano Systems Faculty of Science and Technology University of Twente Enschede The Netherlands; ^3^ Institute of Physical Chemistry University of Münster Münster Germany; ^4^ Catalytic Processes & Materials Group Department of Chemical Engineering Faculty of Science and Technology University of Twente Enschede The Netherlands; ^5^ Department of Chemistry and Nano‐Science Center University of Copenhagen Copenhagen Ø Denmark; ^6^ NNF Quantum Computing Programme Niels Bohr Institute University of Copenhagen Copenhagen Denmark

## Abstract

We report a robust redox‐active N‐heterocyclic carbene (NHC) monolayer that exhibits synapse‐like behavior driven by proton‐coupled electron transfer (PCET). Our quinone‐functionalized NHC (Rex–NHC) forms densely packed, upright self‐assembled monolayers (SAMs) on Au, confirmed by cyclic voltammetry, x‐ ray photoelectron spectroscopy, sum‐frequency generation spectroscopy, and infrared reflection absorption spectroscopy. Molecular junctions built as Au–Rex–NHC//Ga_2_O_3_/EGaIn operate over ± 2 V and can withstand electric fields up to 3.3 GV/m. Bias‐induced PCET toggles between quinone (off) and hydroquinone (on) states, yielding reversible hysteresis with on/off ratios up to 1.9 × 10^2^. The devices exhibit spike‐timing and spike‐rate‐dependent plasticity, demonstrating for the first time molecular‐level neuromorphic behavior using NHCs as anchoring groups.

## Graphical abstract

We report a redox‐active N‐heterocyclic carbene (NHC) monolayer showing synapse‐like behavior via proton‐coupled electron transfer (PCET). These quinone‐functionalized NHCs form dense self‐assembled monolayers and highly stable molecular junctions. Bias‐driven PCET switches quinone/hydroquinone states, producing reversible hysteresis and spike‐timing and rate‐dependent plasticity, demonstrating molecular‐level neuromorphic behavior and associative learning (Pavlov's dog experiment), highlighting the potential of NHC in molecular electronic devices

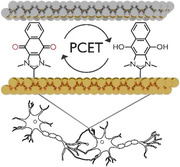

## 1 Introduction

Neuromorphic computing is an information processing approach inspired by natural information processing systems, such as the human brain. It requires new types of adaptive, reconfigurable hardware with time‐dependent switching characteristics [1, 2, 3]. In this context, molecular switches are of interest [2, 4, 5, 6]. Molecular switches have seen advances in photoresponsive and electrochemically‐controlled designs, but robust, reversible switches in molecular‐scale electronic devices remain scarce [7, 8, 9, 10]. The reasons are manifold, as the molecule‐electrode coupling, stabilization of the molecular on and off states, and stability against high electric fields all need to be optimized [11]. For example, the electric fields in molecular devices are on the order of GV/m where charged molecular (redox) states are unlikely to be stable, or spontaneous back switching (due to thermalization, for example), which has to be avoided [12]. In addition, often the stability of the molecule–electrode bond is an issue [13]. In particular, metal‐thiolate bonds have been widely used as molecular anchors to form stable self‐assembled monolayers (SAMs). However, thiol‐based SAMs suffer from limited thermal and oxidative stability [14, 15, 16] and cannot withstand the high electric fields (on the order of GV/m) needed to realize stable switching in tunneling junctions [16, 17].

Recently, N‐heterocyclic carbenes (NHCs) have emerged as a robust alternative, forming highly ordered SAMs with superior chemical and thermal stability and influencing the properties of the material underneath [13, 18, 19, 20, 21, 22, 23, 24, 25, 26, 27, 28, 29, 30, 31, 32]. For example, Cyganik and coworkers have shown that NHC SAMs have excellent thermal desorption energies of 1.98 eV [33]. Despite these favorable properties, electronic devices based on SAMs of NHCs are rare. For example, Venkataraman and coworkers [34] studied single‐molecule NHC junctions with the scanning tunneling microscope‐break junctions (STM‐BJ) technique, Cyganik and coworkers [33], and Yoon and coworkers [35] reported large‐area NHC junctions with the EGaIn technique. These studies neither demonstrated switching nor established the stability of NHC in high electric fields.

Here we report synapse‐like switching behavior by a redox‐active NHC (Rex‐NHC) capable of proton‐coupled electron transfer (PCET). The NHC is functionalized with a quinone/hydroquinone redox‐moiety where tunneling of charge carriers is coupled to proton addition and reversible −OH and C═O bond formation [36, 37]. This PCET feature leads to a molecular system whose conductance states evolve over time and as a function of electrical switching history [38]. We show that it is possible to obtain NHC‐based SAM‐devices that are electrically robust and that can be switched into different memory states, as synapses do, showing spike‐timing and spike‐rate dependent plasticity important for, e.g., Hebbian and associative learning. The junctions have large memory on/off ratios of (1.91 ± 0.30) × 10^2^ at −1.25 V whilst being stable in high electric fields up to 3.3 GV/m. These findings indicate that Rex‐NHC SAMs form dynamic junctions that can emulate synaptic behavior. We believe that our findings will help to explore future studies involving carbenes in molecular devices.

## 2 Results and Discussion

### 2.1 The Molecular Switches

**FIGURE 1:**
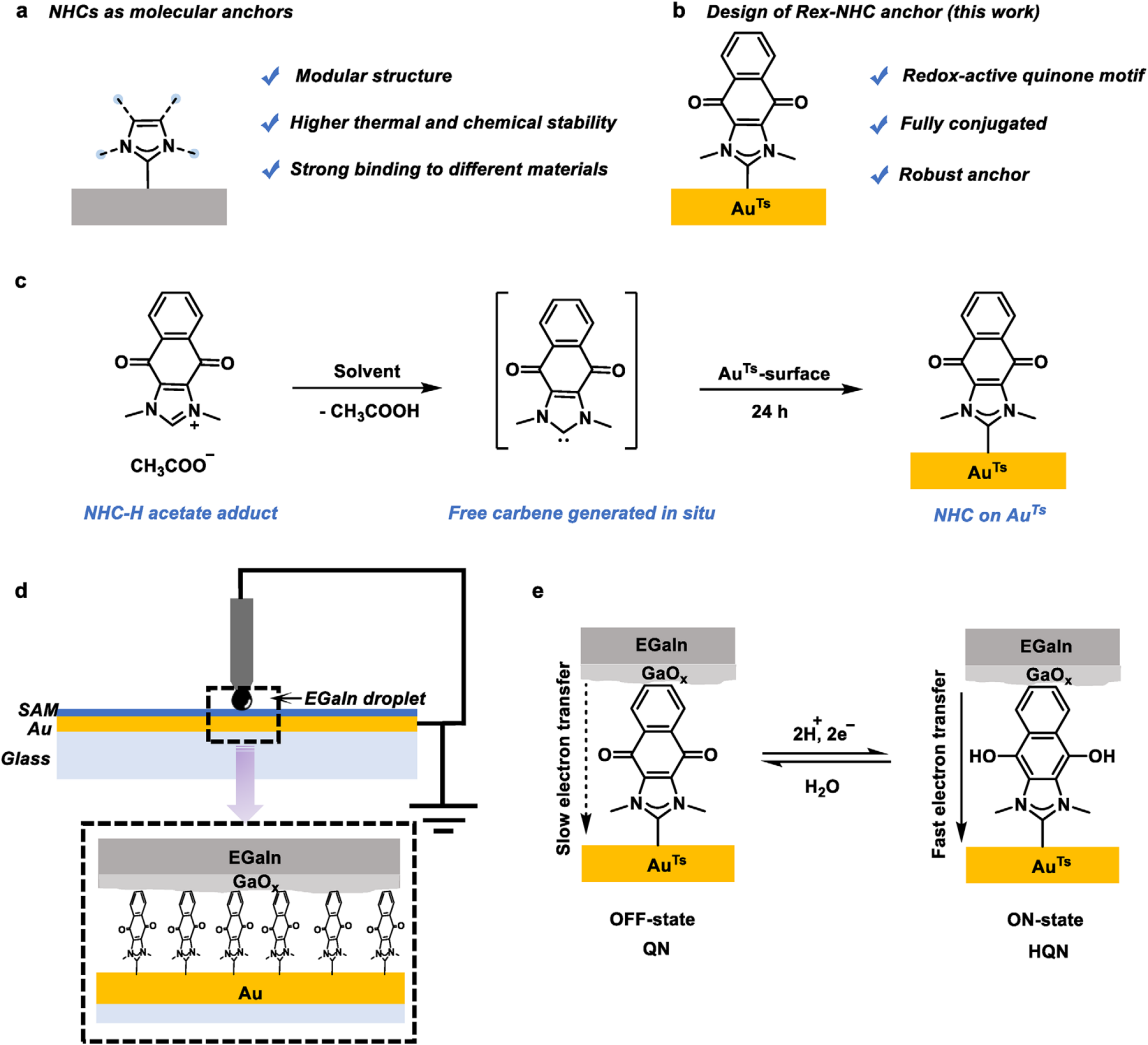
The chemical structure and operating mechanism of the junctions. (a) Advantages of using NHC anchor in molecular junctions; (b) Molecular structure of Rex‐NHCs bound to Au (this work); (c) Preparation method of Rex‐NHC SAMs on Au; (d) Schematic representation of the junction and (e) on and off states as described in the main text.

In this work, NHC‐based SAMs were prepared by immersing freshly template‐stripped gold (Au^TS^) substrates in 1 mM ethanolic solutions of Rex‐NHC acetate for 24 h (see Section S2 and Figure 1c) [39]. We used Au^TS^ surfaces as this method yields clean, ultra‐flat gold surfaces under ordinary laboratory conditions, greatly facilitating the reproducible formation of ordered SAMs [40, 41, 42]. As we show here, the monolayers form spontaneously through the chemisorption of NHC molecules onto Au^TS^, yielding densely packed and well‐defined SAMs. These SAMs were then contacted with EGaIn (eutectic gallium–indium alloy) top electrodes to complete the junctions (Figure 1d), where the EGaIn is a soft, non‐damaging liquid metal electrode commonly used for probing the charge‐transport properties of molecular junctions [43, 44, 45]. Rex‐NHC was selected for its fully π‐conjugated backbone to enhance rigidity, while the NHC anchor provides superior chemical and electrochemical stability compared to conventional thiols [14, 32]. While it is well‐established that quinones (QN) in solution readily undergo two PCET steps to form hydroquinone (HQN) via reversible O─H bond formation [46, 47], we show that Rex‐NHC can also undergo two well‐defined PCET processes whilst immobilized on surfaces. Thus, we conclude that the NHC anchoring group not only firmly binds the molecules to the surfaces but also preserves the well‐defined electrochemical behavior to ensure good switching. Our density functional theory (DFT) calculations support our conclusions and confirm that the junctions are in the on state of the reduced HQN state, leading to a marked increase in current (Figures 1e and 4a). In contrast, the oxidized QN state suppresses the measured currents, switching the junctions off (Figures 1e and 4d). Our results establish that synapse‐like switching can be realized by coupling fast electron transfer to slow proton coupling, in a stable manner, by combining with NHC chemistry.

### 2.2 Monolayer Preparation and Characterization

**FIGURE 2:**
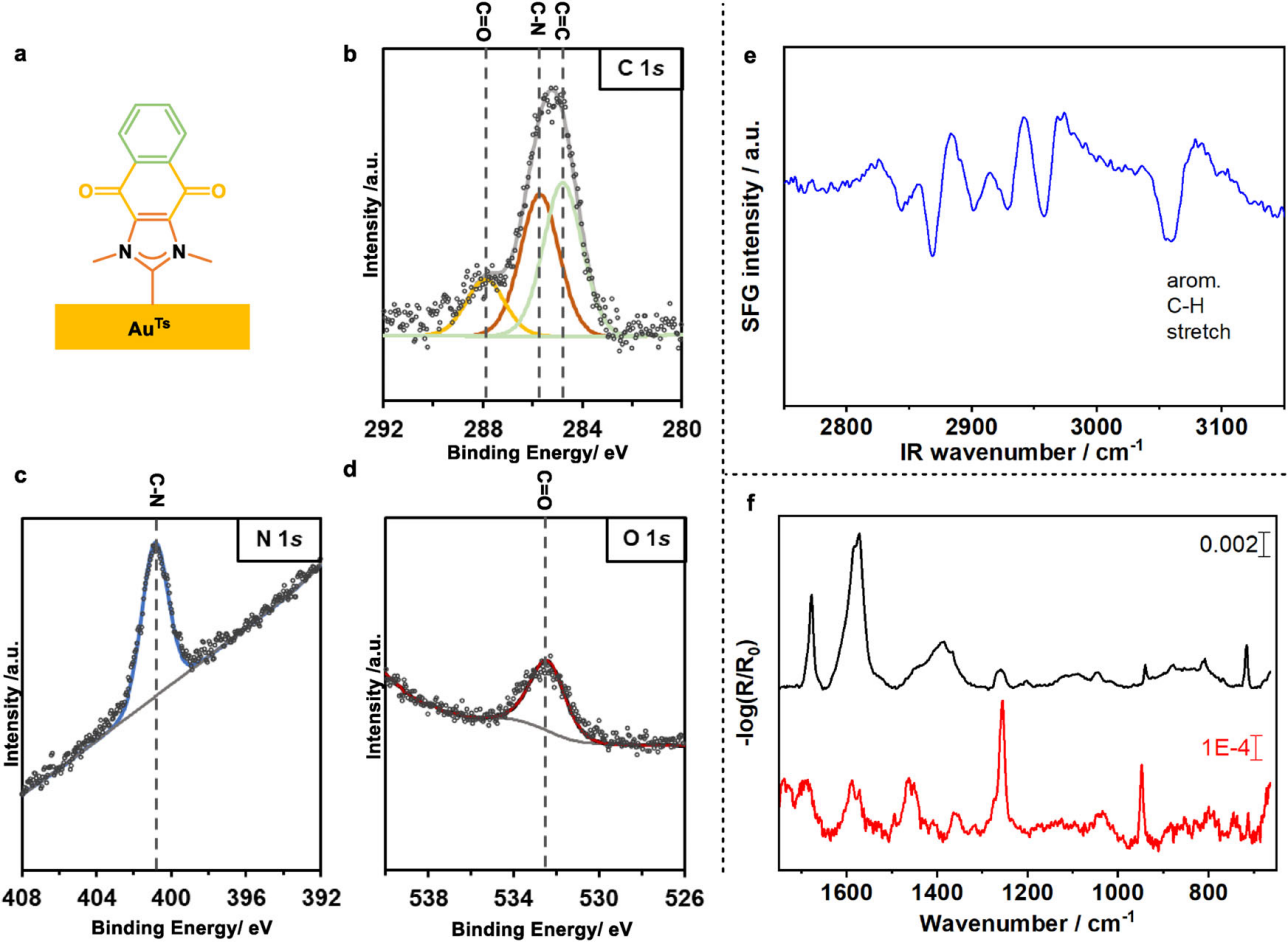
(a) Color‐coded structure of Rex‐NHC on Au; (b) C 1*s*; (c) N 1*s*; (d) O 1*s* XP spectra of Rex‐NHC SAMs on Au^Ts^; (e) SFG spectrum of Rex‐NHC SAM on Au; (f) IRRAS spectrum of 1 mM Rex‐acetate ethanolic solution in a KCl disk (black line) and IRRAS spectrum of Rex‐NHC bound to Au^TS^ (red line).

The synthesis of the Rex‐NHC precursor, SAM formation, and characterization are described in detail in Sections S1–S3, respectively. We characterized the Rex‐NHC SAMs with cyclic voltammetry (CV), X‐ray photoelectron spectroscopy (XPS), sum‐frequency generation spectroscopy (SFG), infrared reflection absorption spectroscopy (IRRAS), and atomic force microscopy (AFM) to establish the surface geometry, electronic structure, topology, and stability of the monolayers. The AFM topography image (Section S3.3) shows that the surface is smooth and has a similar root‐mean‐squared surface roughness as previously reported [28, 35]. We recorded the N 1*s*, C 1*s*, and O 1*s* spectra. The N 1*s* spectra exhibit a single peak centered at 400.9 eV, which is consistent with previously reported values for N 1*s* peaks recorded from other carbene monolayers on Au (see Section S3.1.2) [32, 48]. Figure 2b shows the C 1*s* spectrum which can be deconvoluted through curve fitting to reveal three main components: (i) the peak at ~284.8 eV corresponds to the carbon species of the phenyl ring, (ii) the peak at ~285.7 eV corresponds to carbons directly bonded to nitrogen (i.e., the carbon atoms of the imidazolium ring [48, 49], and (iii) the peak at 287.9 eV corresponds to C═O [50]. The O 1*s* spectrum is dominated by as a single symmetrical peak centered at 532.4 eV corresponding to C═O. We also estimated the surface coverage and the tilt angle using XPS data which is consistent with the coverage values obtained via cyclic voltammetry (Section S3.4). From these data, we conclude that Rex‐NHC forms densely packed monolayers on Au.

To confirm Rex‐NHC forms SAMs with the molecules in the standing‐up orientation, we characterized these surfaces with IRRAS and SFG spectroscopy (Section S3). For IRRAS, we measured the Rex‐acetate 1 mM ethanolic solution clamped between KCl disks and compared it with the spectrum recorded from a Rex‐NHC SAM on Au^TS^ (see Figure 2f). The intensities of the C═O (1678 cm^−1^), N‐CH_3_ (1259 cm^−1^ out of plane ring bending (716 cm^−1^) were used to establish the orientation of Rex‐NHC moieties in the SAM (Section S3.2) [51, 52] because these vibrations correspond to dipole transitions along the three orthogonal axes relative to the gold surface (x, C═O; y, N‐CH_3_; z, out of plane). The strong intensity of the N─CH_3_ bend at 1259 cm^−1^, and the attenuated C═O stretch at 1678 cm^−1^ are consistent with an upright orientation of the Rex─NHC monolayer on the surface [52]. Figure 2e shows the SFG spectroscopy results around the aromatic C─H stretching band at 3070 cm^−1^ which was selected as its dipole moment lies in plane of the aromatic ring making this mode a suitable probe for the orientation of Rex‐NHC on the Au surface as follows: [53, 54] in case of flat lying Rex‐NHC, the aromatic CH is dipole‐forbidden and thus SFG inactive, but the opposite is true for an upright orientation. The pronounced SFG intensity of the aromatic CH stretching mode at 3070 cm^−1^ (Figure 2e) supports our conclusion that the Rex‐NHC molecules form upright standing SAMs [55].

### 2.3 Proton‐Coupled Electron Transfer

**FIGURE 3:**
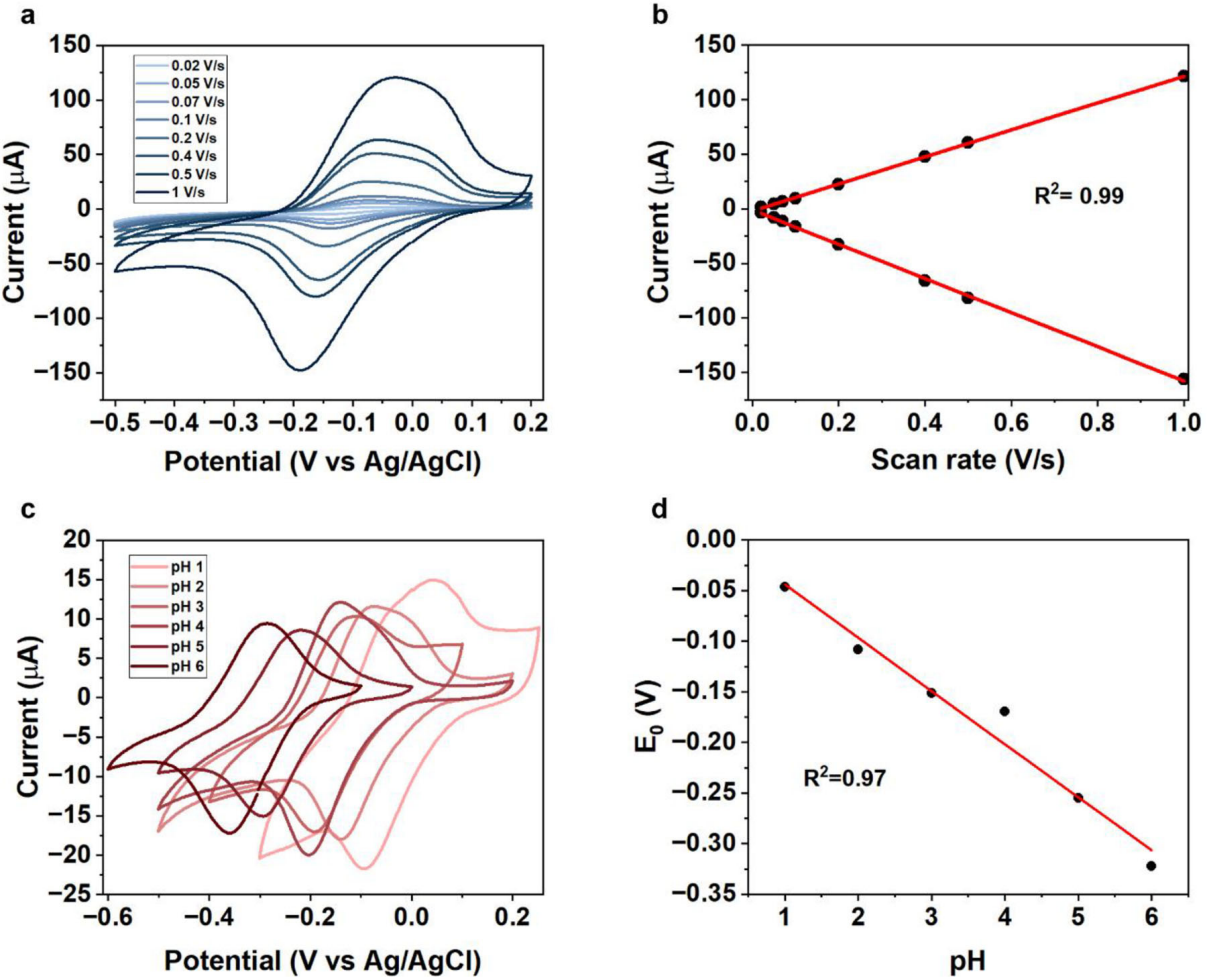
(a) Cyclic voltammograms of Rex SAM on Au in 0.1 M NaClO_4_ at pH 2 with Ag/AgCl as the reference electrode and a Pt mesh counter electrode recorded for *ν* = 0.020–1.0 V/s; (b) Plot of the anodic and cathodic current as a function of *ν*. R^2^ is the correlation coefficient; (c) Cyclic voltammogram recorded at 0.1 V/s of the Rex SAM recorded for pH = 1–6 at intervals of 1; (d) Plot of formal potential (*E°*) versus pH.

The cyclic voltammograms (CVs) were recorded in aqueous 0.1 M NaClO_4_ electrolyte adjusted to pH = 2 with HClO_4_ (while keeping the total concentration of ClO_4_ at 0.1 M) at scan rates from 0.02 to 1 V/s (Figure 3a). The CV is dominated by a broad redox wave similar to previously reported CVs of quinone SAMs in aqueous electrolyte, which is ascribed to conversion from the quinone to the hydroquinone [46, 47, 55]. The positions of the anodic (*E*_a_) and cathodic (*E*_c_) peak potentials show a small potential shift, resulting in a reduction of Δ*E*_p_ of 108 mV. The peak is broad, with a full width at half maximum of 164 mV (Table S3). The cathodic (*I*_pc_) and anodic (*I*_pa_) peak currents increase linearly with *ν* (Figure 3b), characteristic of a surface‐confined redox process. The electrochemical formal potential *E* ^0^ around −0.11 V versus Ag/AgCl is also in close agreement with thiols‐based quinones SAMs [46, 55]. These observations are characteristic of a two‐electron reduction process involving both quinone/hydroquinone units, as previously reported for thiol‐based quinones [46, 55]. Interestingly, despite the conjugated nature of the quinone, the overall redox response is very similar to that of alkanethiolate SAMs with quinone/hydroquinone terminal group units, indicating weak electronic coupling with the electrode (which is in agreement with current‐voltage characteristics described below and from earlier reports) [56].

The surface coverage of the Rex SAM (Γ_Rex_) was estimated by integrating the cathodic peak to determine the total charge (Section S3.5) [57]. We found an estimate of the surface coverage of Rex‐NHC SAMs of (3.86 ± 0.11) × 10^−10 ^mol/cm^2^ (2.3 ± 0.07 molecules/nm^2^), indicating the formation of a densely packed monolayer on the gold surface. To confirm the redox mechanism is PCET, we measured cyclic voltammograms at 0.1 V/s for different pH values between 1 and 6 using 0.1 M NaClO_4_ aqueous electrolytes, where the pH was adjusted with HClO_4_. Figure 3c shows the CVs recorded from 0.25 to −0.6 V, and CVs shift cathodically. Figure 3d shows the linear relation of *E* ^0^ versus pH with a slope of 53 ± 4 mV/pH, which is close to the value of 59 mV/pH for a PCET mechanism [58]. From these measurements, we conclude that the quinone functionality of our carbene‐immobilized on Au can be converted to its hydroquinone form by PCET [59].

### 2.4 J(V) Measurements and Mechanism

**FIGURE 4:**
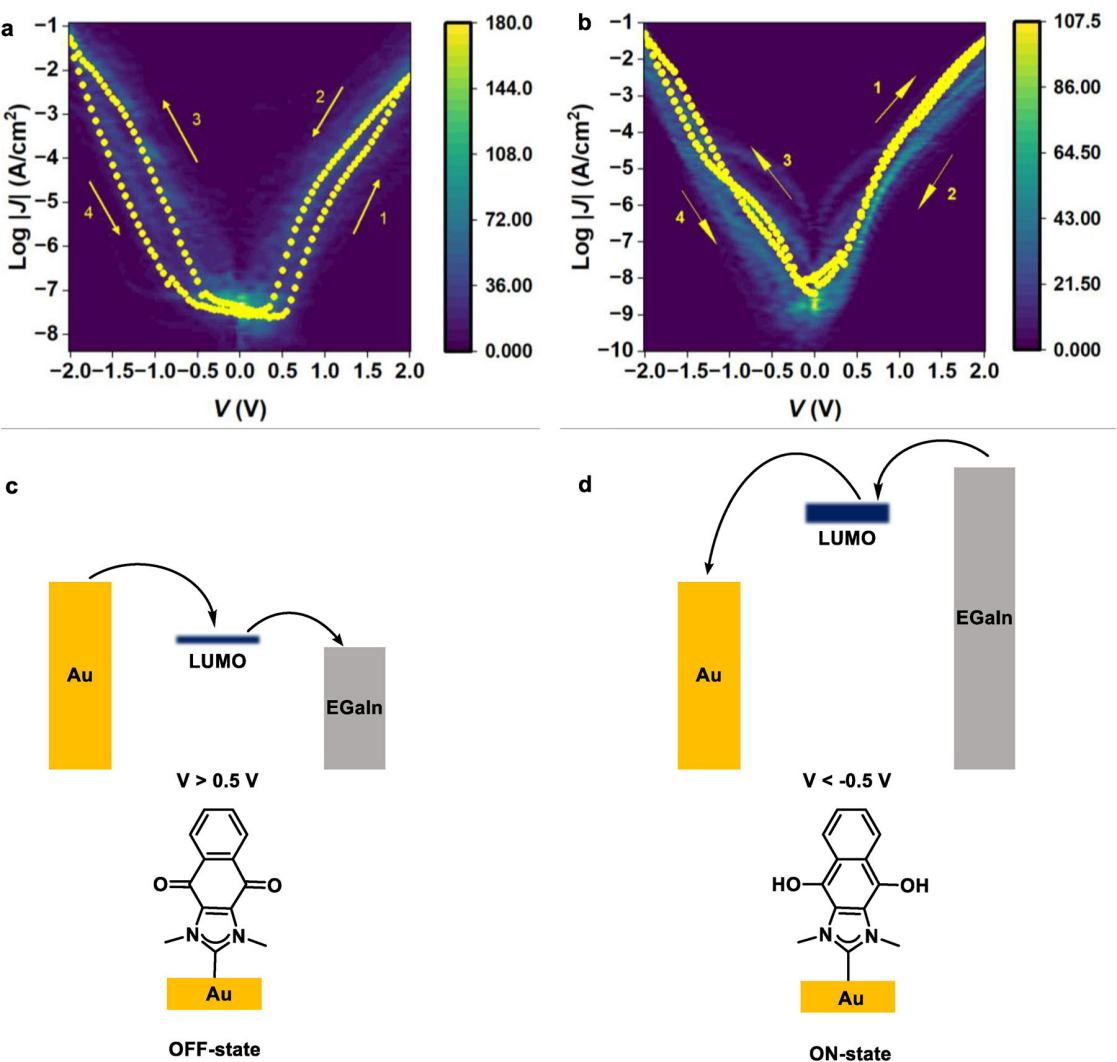
(a) Heatmap of log_10_|*J*| versus* V* at 40% relative humidity. (b) Heatmap of log_10_|*J*| vs. *V* at 1% relative humidity. The yellow dots are the Gaussian log‐average curve (<log_10_|*J*|>_G_ vs. *V*). Energy level diagrams of the off (c) and on (d) states of the junction, where tunnelling is mediated by the LUMO indicated in blue. The width of the LUMO indicates the coupling strength as derived from DFT.

We measured the current (*J* in A/cm^2^) as a function of voltage *V* (in V) of the Au─Rex‐NHC//Ga_2_O_3_/EGaIn junctions for *V* = ± 2.0 V. We used a statistically large number of *J*(V) traces of *N* = 450 scans in our analysis, which are all shown in the heat map of Figure 4a. From these curves, we established the Gaussian log‐average of all currents <log_10_|*J*|>_G_ measured for each applied *V*, resulting <log_10_|*J*|>_G_ versus *V* curves, along with log‐standard deviations, as shown in Figure S7b following previously reported procedures (Section S4) [60]. The arrows indicate the direction of bias, where one voltage cycle was 0 V → 2.0 V → 0 V → −2.0 V → 0 V. The yields of working junctions are close to 90%, comparable to values reported previously for related systems such as the BIMe‐NHC junctions reported by the Cyganik group [20, 33]. The most striking feature is the large ambipolar hysteresis with an on/off current ratio at *V* = −1.25 V of (1.91 ± 0.30) × 10^2^ and at *V* = 0.55 V of (1.57 ± 0.31) × 10^2^. A small current rectification of 7.7 ± 2.2 at ± 2.0 V is also observed, which indicates a small asymmetry in the junction, probably due to the asymmetrical nature of the molecule or different binding chemistries with the bottom (covalent) and top (van der Waals) electrodes.

We explain this memory behavior as follows. By starting the voltage sweep at 0 V to + 2 V, Rex‐NHCs are reduced from QN to the HQN state, turning the junction on. So at 0 V, the junctions start in their QN off‐state (due to its narrow LUMO and poor alignment with the Fermi level as described in the theory section below), indicated by arrow 1, but return to 0 V in their on‐state, indicated by arrow 2 (Figure 4a). At positive bias, the molecules are reduced from the QN to the HQN on‐state (due to broadening of the LUMO and more favorable energy‐level alignment, see below). The junctions turn off at opposite bias (arrow 3) and are oxidized back to QN off‐state, resulting in a lower current when the voltage is swept back from −2 to 0 V (arrow 4). The hysteresis observed originates from PCET and dynamic covalent bond formation, which stabilize the on/off states. To prove the PCET is the underlying driving mechanism for switching, we repeated the experiment, but this time under dry conditions ( < 10 ppm water). Figure 4b shows that the hysteresis disappears, confirming that a proton source from water in air is essential, as expected for the proposed PCET mechanism outlined in Figure 1.

Remarkably, Rex‐NHC SAMs can withstand a bias voltage of up to ± 2 V, corresponding to an electric field strength of 1.7 GV/m. Therefore, we determined the breakdown voltage *V*_bd_ (in V) as follows. We first formed a junction with *J*(V) characteristics that fall within the σ_log_ recorded for *V* = ±2 V. Then, the bias was ramped to ± 5 V until a sudden current increase indicated breakdown. This was repeated 40–50 times in each bias direction, leading to *E*_bd_ = 3.3 GV/m at positive bias and *E*_bd_ = 2.7 GV/m at negative bias (Figure S8a,b). These measured values of electrical breakdown are comparable with Au‐SC_18_//Ga_2_O_3_/EGaIn molecular tunneling junction (*E*_bd_ = 1.3 GV/m), which are twice the thickness of Rex‐NHC SAMs [61]. To the best of our knowledge, previous studies on tunneling junctions based on NHC monolayers reported have only examined a bias window of ± 1 V [33, 34, 35, 62], but our experiments show that these SAMs are very stable. Further, these SAMs were stored in ambient conditions for 2 months and showed a stable on/off ratio over that time period (Figure S8c). For the sake of completion, retention and endurance tests are given in Figure S8d,e. The endurance test reflects the dynamic behavior of the junctions for the first 60 switching cycles, after which the on/off ratios of the junctions stabilized. Similarly, the on/off ratio decreases in the 100 s of the retention test but then remains stable for almost 1500 s.

### 2.5 Theoretical Model

**FIGURE 5:**
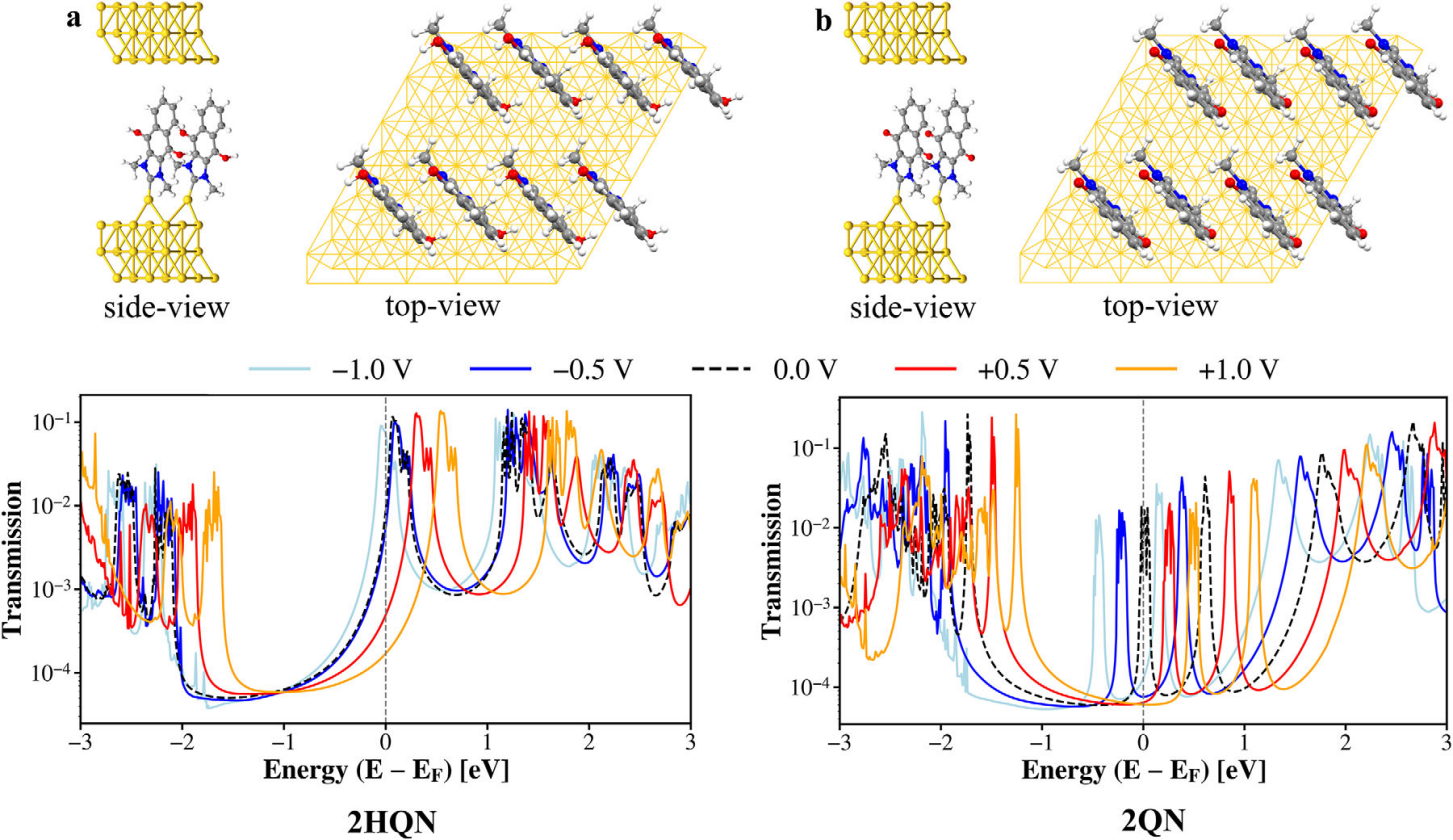
Side‐and top‐view of the optimized structures of Au‐2NHC‐Au junction for both (a) HQN and (b) QN forms. H, C, N, O, and Au are represented by white, grey, blue, red, and yellow colored balls, respectively. (Top panel) Calculated transmission function per molecule for Au‐2NHC‐Au junction as a function of energy relative to the Fermi energy (*E*_F_) for both HQN and QN form. (Bottom panel)

To elucidate the origin of the high on/off current ratios observed under both positive and negative bias, we computed the transmission functions of the NHC‐based SAM junctions at finite bias for both QN and HQN states at two packing densities (Au− *n*NHC− Au, where n  = 1 or 2) using DFT and nonequilibrium Green's function (NEGF) methods [63, 64]. Computational details are provided in the SI. The results for the higher packing density (n  =  2) are shown in Figure 5, while those for the lower density (n  =  1) are provided in Figure S9. The transmission plots reveal that electron transport is predominantly LUMO‐mediated, as the LUMO resonance lies closest to the Fermi level in both cases (i.e., higher and lower packing density). A broader LUMO peak is seen for the HQN, and in contrast, a sharper LUMO peak is seen for the QN form for both higher and lower density packing. This indicates that the PCET (Figure 1) mediates switching between the on and off states by the changes in the molecule electrode coupling strength (or broadening of the LUMO level) as illustrated in the energy level diagrams in Figure 4. Our calculations show that for higher packing density, enhanced π− π stacking between adjacent NHC molecules leads to an increase in transmission per molecule. This is attributed to a shift of the LUMO‐derived peak closer to the Fermi level for higher packing density compared to the sparsely packed junction (see Figures 5 and S9). The π–π stacking enhanced transmission has also been reported in similar π‐conjugated N‐containing heterocyclic molecules [65]. Given that electron transport is LUMO‐dominated, it is worthwhile to analyze how inter‐orbital coupling between adjacent NHC molecules affects the LUMO energy levels. To quantify this interaction, we computed the LUMO energies of NHC dimers extracted from the optimized junction geometries. The individual LUMO energies of each NHC, as well as the LUMO and LUMO+1 energies of the resulting dimer, were evaluated for both redox states, QN and HQN, as shown schematically in Figure S10. The QN dimer exhibits a LUMO‐LUMO+1 separation of ∼0.6 eV, while the HQN dimer shows a much smaller splitting of ∼0.1 eV, indicating a significantly stronger LUMO‐LUMO interaction in the QN form (Figure S10). In contrast, the HQN‐based junctions have weaker coupling, leading to closely spaced LUMO and LUMO+1 orbitals, which merge into a broader single peak (broader relative to the QN form) in the transmission plot. Consistent results are obtained from the full junction orbital analysis, where the energy splitting between NHC‐localized LUMO and LUMO+1 remains larger in the QN system (ca.∼0.55 eV) compared to the HQN system (ca.∼0.06 eV) (see Table S5).

Applying increasingly negative bias shifts the sharp NHC‐localized LUMO peak of QN significantly to lower energies, while HQN shows only a minor shift for both packing densities (see Figure 5 and Figure S9). Consequently, HQN maintains higher transmission under negative bias, whereas QN transmission decreases. Under positive bias, both LUMOs of QN and HQN shift towards higher energy, reducing transmission around the Fermi level. However, this reduction is more pronounced for the QN form compared to the HQN form at higher packing density because of the narrow, sharply peaked LUMO‐derived transmission of QN that shifts away from the Fermi level under applied bias, leading to a rapid decrease in transmission. In contrast, a broader LUMO‐derived transmission of the HQN retains its transmission probability around the Fermi level despite energy shifts induced by bias. The distinct bias responses of QN and HQN result in different transmissions around the Fermi level under both positive and negative bias. At higher bias magnitudes, the HQN junction consistently exhibits higher transmission in the bias window $\;(E_{F}\pm \frac{eV}{2})$, acting as the “on” state, while the QN junction shows suppressed transmission in the bias window and functions as the “off” state. This bias‐dependent contrast results in pronounced on/off ratios seen in the experiment (see Table S6).

### 2.6 Emulating Synapse Plasticity

Short‐term synaptic plasticity in biological systems arises from the dynamic availability of neurotransmitters, which determines whether successive stimuli lead to enhanced (e.g., paired pulse facilitation, PPF) or diminished (e.g., paired pulse depression, PPD) postsynaptic responses. Facilitation typically occurs when stimuli are spaced sufficiently to allow recovery, while high‐frequency inputs can deplete active signaling components, resulting in depression [59].

**FIGURE 6:**
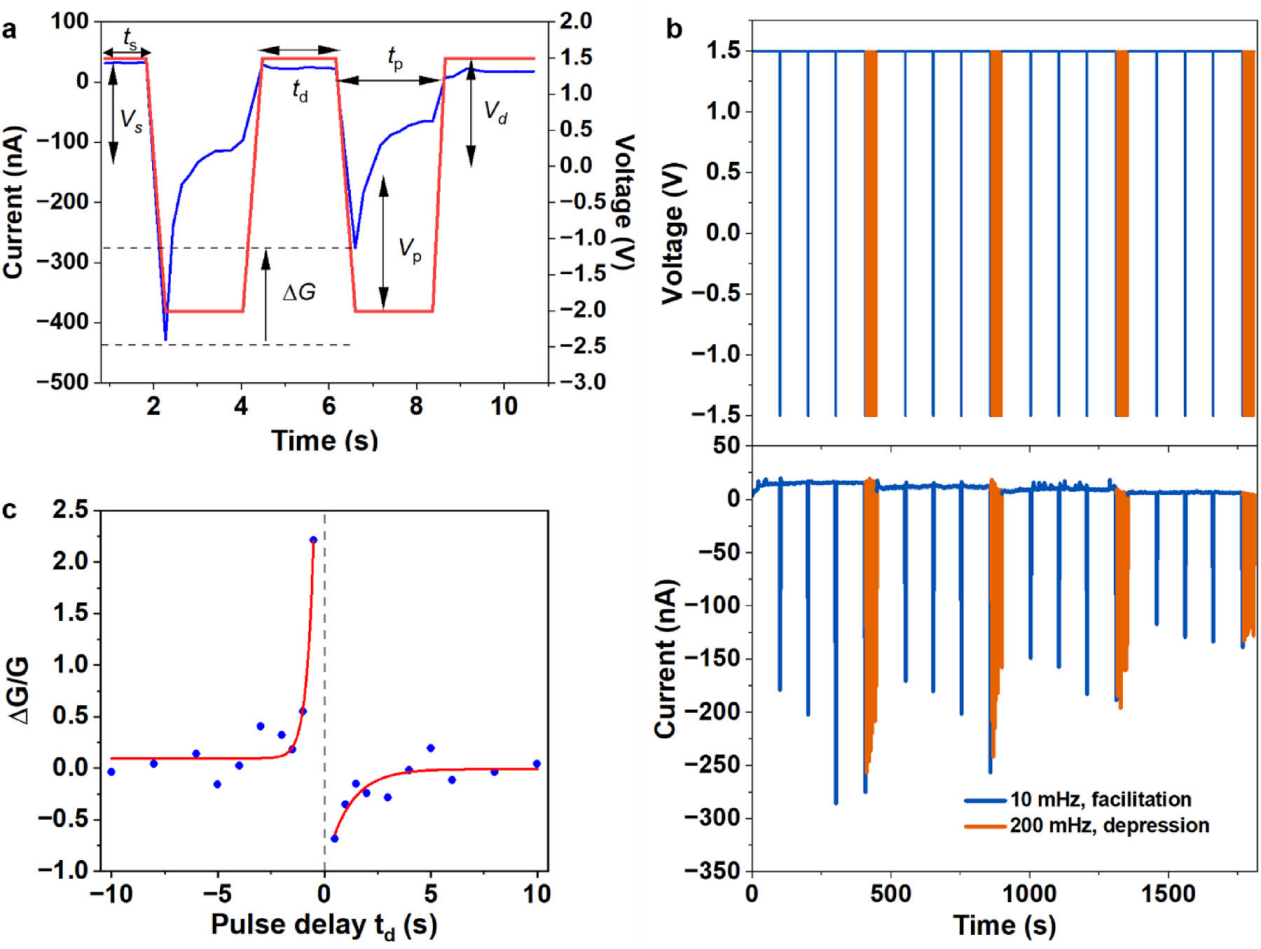
(a) Typical voltage‐paired‐pulse sequence and current response. ∆*G* is the difference in chordal conductance between the measured and reference pulse as indicated by the arrow. Also pulse delay *t*_d_, voltage pulse *t*_p_, set time *t*_s_, amplitude voltage *V*_p_, set voltage *V*_s_ function of pulse duration are defined; (b) voltage sequence used to emulate spike rate dependent plasticity and pulse sequence exhibiting spike‐rate‐dependent plasticity with *V*_p_ =  −1.5 V at 10 mHz or 200 mHz and *V*_r_  = 1.5 V; (c) ∆*G*/*G* measured as function of interpulse delay with corresponding fits (red lines).

To demonstrate PPD and PPF, we used the normalized change in chordal conductance between pulse pairs, *ΔG/G*, to define changes in “synaptic weight”, as defined in Figure 6a. Figure 6a defines the voltage pulse‐pair protocol and the corresponding current output used to extract *ΔG/G*. Two identical programming pulses of amplitude *V*_p_ and duration *t*_p_ are applied and separated by an interpulse delay *t*_d_. The current response to each pulse is converted to the chordal conductance (*G* = *I*_bias_/*V*), where we denote the conductance associated with the first pulse as *G*_ref_ (reference) and that of the second pulse as *G*_meas_ (measured). The paired‐pulse plasticity is then calculated as *ΔG/G* = (*G*_meas_−*G*_ref_)/*G*_ref_. A positive Δ*G*/*G* indicates facilitation, whereas a negative *ΔG/G* indicates depression. Between a pulse pair, we applied a set voltage (*V*_s_) to bias the junction toward facilitation or depression using *V*_s_ = + 1.5 V for a set time *t*_s_ = 3 s, after which the next pulse was applied. First, we discuss spike rate‐dependent plasticity (SRDP) shown in Figure 6b. Here, we used a low and high frequency pulse train by varying *t*_s_ = 99 s and *t*_s_ = 4 s (at V_s_ = + 1.5 V) while *t*_d_ = 1 s (at *V*_p_ = −1.5 V and *t*_p_ = 1 s) leading to the frequencies of 10 mHz (blue in Figure 6b) and 200 mHz (orange in Figure 6b). The top panel of Figure 6b shows the voltage pulse sequences, and the bottom panel shows the corresponding current sequences. In this experiment, *V*_s_ facilitates the junctions to HQN state, while *V*_p_ depresses the junctions to QN state (see Figure 1e). Therefore, the competition between facilitating the junction and cumulative back switching during the different pulse trains determines the plasticity. Figure 6b shows that for the 10 mHz pulse trains yield net facilitation, whereas 200 mHz trains yield net depression.

To demonstrate spike‐timing‐dependent plasticity (STDP), where the order in which the pulses are applied and the delay between them modulates Δ*G/G*, we applied pulses pairs of *V*_p_ = −2.0 V and *t*_p_ = 1 s followed by a varying delay time *t*_d_ at voltage delay *V*_d_ = + 1.5 V. Between two consecutive pulse pairs, we applied *V*_s_ = + 1.5 V for 3 s. Figure S12 shows the complete voltage trains (and current response) for all values of *t*_d_ where each pulse pair is indicated by a color code. For each pulse pair, we determined the Δ*G/G* values *t*_d_ > 0 and *t*_d_ < 0, whereby a positive (negative) value of *t*_d_ signifies that the reference pulse occurs before (after) the measured pulse. Figure 6c shows the results from which we conclude that the junctions show STDP. To extract the characteristic decay time *τ*, we fitted the data to *I*  (*t*) = *y*_0_  + *A*_1_ *e* ^−*t*/τ^ (red solid lines Figure 6c) to give *τ* = 1.2 s for positive *t*_d_ and 0.4 s for negative *t*_d_. These results demonstrate that, as in biological neural systems, the frequency and the timing of the signal decide the (synaptic) junction response.

### 2.7 Pavlov Learning

**FIGURE 7:**
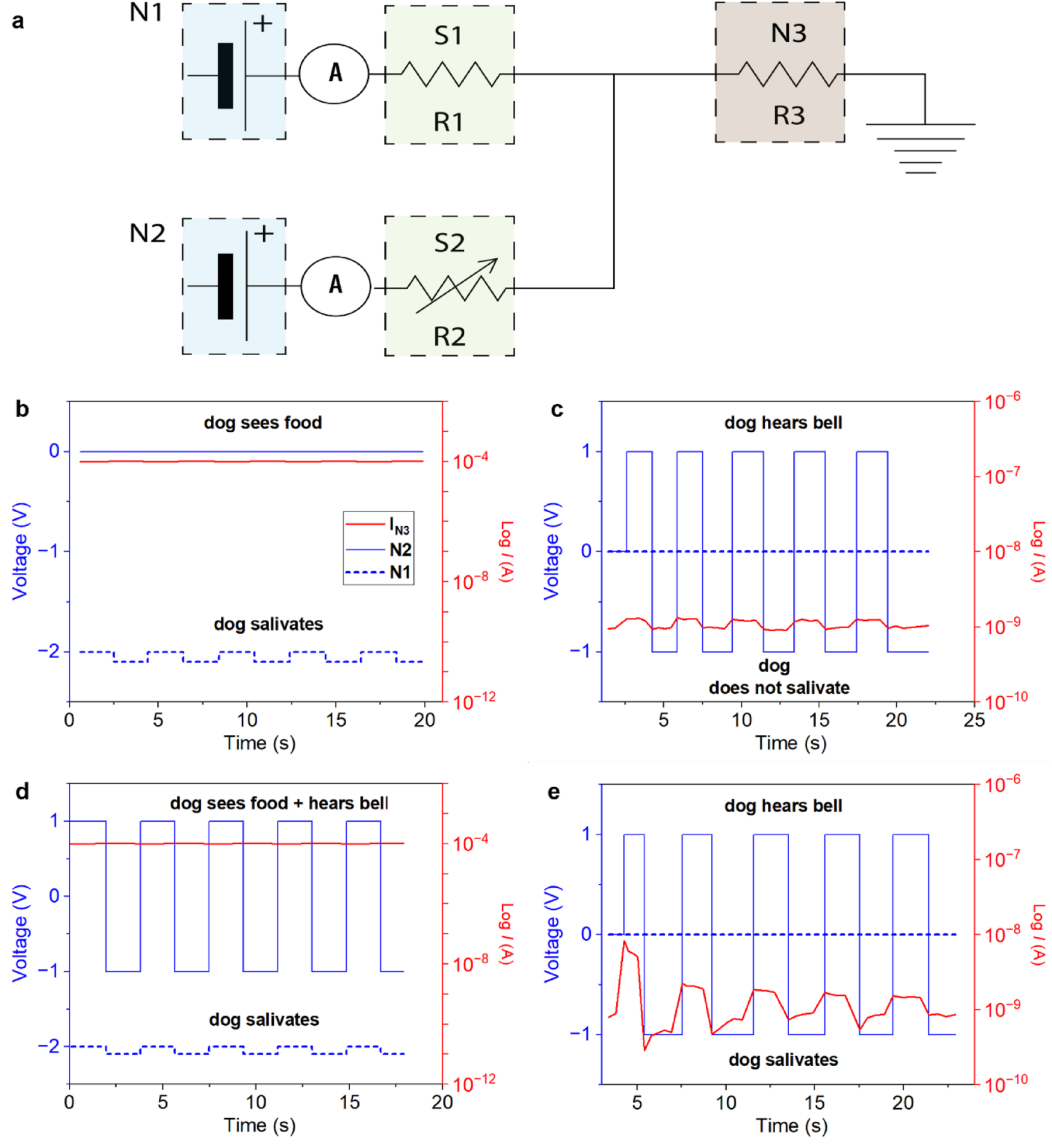
(a) Schematic of the electrical circuit. Two neurons, N1 and N2, are connected to an output neuron, N3(R3), via a fixed resistor (S1, R1) and the adaptive junction (S2, R2). (b–e) Conditioning protocol for four consecutive phases. (b) N1‐only stimulation (“dog sees food”) gives unconditional output, (c) N2‐only stimulation (“Dog hears bell”) is initially inactive, (d) Simultaneous N1 and N2 stimulation (“Dog sees food and hears bell”) trains S2. (e) N2‐only stimulation after training yields an output (“Dog hears bell→salivation”)

As a demonstration of the potential of our junctions, we simulated Pavlov's dog test as follows. Figure 7 shows the electronic equivalent scheme of Pavlov's dog experiment (see Section S7 for details), illustrating how a trainable synapse can implement Hebbian learning, i.e., the training of one input through its simultaneous operation with another (“neurons that fire together, wire together”). Two source meters (neurons N1 and N2) deliver voltage pulse trains (fire) to synapse S1 materialized by a low ohmic resistor (R1 = 10 kΩ), and a trainable synapse S2 (R2, Rex–NHC SAM junction). Their common node is coupled to a low‐ohmic resistor (R3 = 10 kΩ) acting as the output neuron (N3). The output current I_N3_, which is the equivalent of the post‐synaptic current, is indirectly measured at N1 by a source meter (Figure S13). The conditioning protocol comprises four consecutive phases. “Dog sees food” is represented by N1‐only stimulation (Figures 7b and S13a for the equivalent circuit), where N1 produces an output at N3. N2‐only stimulation is the equivalent of “Dog hears bell”, where the output at N3 remains negligible because S2 is not trained yet (Figure 7c). Importantly, the N2‐voltage window is chosen such that the Rex‐NHC junction is close to activation. Simultaneous firing of N1 and N2 (“Dog sees food and hears bell”), sensitizes S2 (training) in the N2 channel (Figure 7d). Figure 7e shows N2‐only stimulation after training, where the same N2 pulse train now produces a clear output at N3, demonstrating associative learning (see Figure S13b,c for the equivalent circuit and the full sequence, respectively).

## 3 Conclusions

We have fabricated SAMs of Rex‐NHC and characterized their structure and electrochemical behavior, demonstrating stable redox switching by proton‐coupled electron transport. These Rex‐NHC SAMs yield stable molecular electronic junctions that are stable in high electric fields up to 3 V/m. By incorporating quinone/hydroquinone redox functionality, we demonstrate that the charge transport and memory switching mechanism relies on proton‐coupled electron transport, where water from air is the proton source. The junctions have large on/off ratios of two orders of magnitude in both bias directions. These values, combined with the high stability of these junctions, are remarkable considering that the thickness of the molecular layer is 1.2 nm. Since fast electron transfer is coupled to slow proton coupling, the junctions show time‐dependent switching behavior similar to that of synapses. We demonstrate spike‐timing and spike‐rate dependent plasticity, a key feature associated with synaptic switching, forming the basis for potential use in spike‐timing dependent neuromorphic computation schemes. Our results demonstrate the potential of NHCs in molecular‐scale electronics to fabricate functional devices that are stable against high electric fields and aging.

## Conflicts of Interest

The authors declare no conflicts of interest.

## Supporting information




**Supporting File 1**: anie72138‐sup‐0001‐SuppMat.Docx.

## Data Availability

The data that support the findings of this study are available from the corresponding author upon reasonable request.
