# Supplementary material for: Stable Synapse‐Like Memory Switching in N‐Heterocyclic Carbene Monolayers
Source: Angew Chem Int Ed Engl. 2026 Apr 17;65(25):e1823213. doi: 10.1002/anie.1823213 (PMC13266966; doi:10.1002/anie.1823213)
Supplement: Supplementary file 1 — Supporting File 1: anie72138‐sup‐0001‐SuppMat.Docx. [file ANIE-65-e1823213-s001.docx]

Supporting Information

Stable Synapse-Like Memory Switching in N-Heterocyclic Carbene Monolayers

*Ankita Das^[a]†^, Alessandro Borrini^[b]†^, Christian Gutheil^[a]^, Björn Braunschweig^[c]^, Billura Shakhayeva^[c]^, Georgios Katsoukis^[d]^, Ab F. Nieuwenhuis^[b]^, Raka Ahmed^[e]^, Susanne Leitherer^[e]^, Gemma C. Solomon^[e,f]^*, Frank Glorius^[a]^*, Christian A. Nijhuis^[b]^**

† These authors equally contributed to this work

[a] Organisch-chemisches Institut, Corrensstraße 40, 48149, Münster, Germany

[b] Hybrid Materials for Opto-Electronics Group, Department of Molecules and Materials, MESA+ Institute for Nanotechnology, Molecules Center and Center for Brain-Inspired Nano Systems, Faculty of Science and Technology, University of Twente, P.O. Box 2017, 7500 AE Enschede, The Netherland

[c] Institute of Physical Chemistry, University of Münster, Corrensstraße 28/30, 48149 Münster, Germany

[e] Catalytic Processes & Materials Group, Department of Chemical Engineering, Faculty of Science and Technology, University of Twente, P.O. Box 2017, 7500 AE Enschede, The Netherland

[e] Department of Chemistry and Nano-Science Center, University of Copenhagen, Universitetsparken 5, DK-2100, Copenhagen Ø, Denmark.

[f] NNF Quantum Computing Programme, Niels Bohr Institute, University of Copenhagen, DK-2100 Copenhagen, Denmark

**Contents**

[1. Synthesis 3](#_Toc223004942)

[**1.1 General Information** 3](#_Toc223004943)

[**1.2 Synthesis of redox-active NHC-acetate** 4](#_Toc223004944)

[**1.3 NMR Spectra** 7](#_Toc223004945)

[**1.4 Direct-inlet spectra of Rex(H)-CH_3_CO_2_ precursor** 9](#_Toc223004946)

[2. Self-assembled monolayer formation on Au^TS^ with NHC 9](#_Toc223004947)

[**2.1 Au^TS^ surface preparation** 9](#_Toc223004948)

[**2.2 Modification with NHCs** 10](#_Toc223004949)

[3. Surface characterization 10](#_Toc223004950)

[**3.1 X-ray photoelectron spectroscopy (XPS) measurements** 10](#_Toc223004951)

[**3.1.1 NHC identification** 10](#_Toc223004952)

[**3.1.2 Experimental estimation of surface coverage** 12](#_Toc223004953)

[**3.1.3 XPS spectra of Rex-NHC@Au** 15](#_Toc223004954)

[**3.2 IRRAS** 15](#_Toc223004955)

[**3.3 Atomic force microscopy** 16](#_Toc223004956)

[**3.4 Sum-frequency generation (SFG) spectroscopy** 17](#_Toc223004958)

[**3.5 Electrochemistry** 17](#_Toc223004959)

[**3.6 Uv-vis spectroscopy** 19](#_Toc223004960)

[4. EGaIn J-V measurements 20](#_Toc223004961)

[**4.1 Voltage breakdown measurements, aging, retention and endurance** 21](#_Toc223004962)

[5. Computational Details 23](#_Toc223004963)

[6. Synaptic plasticity 27](#_Toc223004964)

[7. Pavlov learning 27](#_Toc223004965)

[8. References 30](#_Toc223004966)

# **1. Synthesis**

## **1. 1 General Information**

All reactions were carried out in oven-dried glassware with oven-dried Teflon-coated magnetic stir bars. Dry solvents were either taken from a solvent purification system (HPLC grade, dried over activated alumina columns) or purchased from Acros Organics, Sigma-Aldrich or Carl Roth (stored over activated molecular sieves). All reagents were obtained from ABCR, Acros Organics, Alfa Aesar, Carbolution Chemicals, Carl Roth, Chempur Combi-Blocks, Fisher Scientific, Fluorochem, Merck, Sigma-Aldrich, TCI Europe or VWR and utilized as received.

**1H- and 13C-NMR spectra** were recorded on a Bruker AV 400 at room temperature. Chemical shifts (δ) were given in ppm. The residual solvent signals were used as references and the chemical shifts converted to the TMS scale (MeOD: δH = 4.87 ppm, δC = 49.00 ppm; CD_2_Cl_2_: δH = 5.32 ppm, δC = 54.00 ppm; D_2_O: δH = 4.79 ppm,). All the NMR spectra were processed using Mestrenova 14 applying standard phase and baseline corrections. Coupling constants (J) are quoted in Hz.

**High resolution mass spectra (HRMS)** were recorded on a Thermo Scientific Exploris 120 Electrospray Orbitrap in electrospray ionizsation mode (ESI). ESI spectra show relative abundance after normalization against maximum signal intensity level (NL) in dependence of m/z.

**Direct inlet electron impact mass spectra with temperature profile (Direct inlet EIMS)** were performed on a Thermo Scientific TSQ 7000 with a pressure of 1.99 to 2.66 × 10^-7^ mbar. The ionization mode was electron ionization with an electron acceleration of 70 eV. The cathode was cooled by an internal water-based cooling system to around 32 °C at the beginning of the experiment and heated up consequently to around 250 °C. The solid material was placed in a clean crucible, transferred into the high vacuum of the spectrometer and heated from room temperature to 400 °C to follow the evaporation of the “free” NHC.

## **1.2 Synthesis of redox-active NHC-acetate**

**Figure S1**: Synthesis of Redox-active acetate [ReX(H)CH_3_CO_2_]

**2,3-diaminonaphthalene-1,4-dione (1)**

2,3-dichloro-1,4-naphtoquinone (2.26 g, 10.0 mmol, 1.0 equiv) was dissolved in CH_3_CN (46 mL) and potassium phtalimide (3.88 g, 21.0 mmol, 2.1 equiv) was added to the mixture. The solution was refluxed under argon for 16 h. After cooling to room temperature, yellow solid was collected by filtration and further dried under vacuum. The dried yellow solid was suspended in distilled water (50 mL) and 2.70 mL of hydrazine hydrate was added. After reaction was stirred overnight at 60 °C, a dark blue-violet powder was formed. This solid was collected after filtration and washing with distilled water. After drying the desired compound is obtained. (1.62 g, 86.17 mmol, 86 % yield)

**^1^H NMR** (400 MHz, DMSO) 𝛿 (ppm): δ 7.8 – 7.7 (m, 2H), 7.6 – 7.5 (m, 2H), 5.4 (s, 4H).

**^13^C NMR** (101 MHz, DMSO) 𝛿 (ppm): δ 178.4, 132.6, 131.1, 127.6, 124.6.

**HRMS (ESI+):** m/z calculated for C_10_H_8_N_2_O_2_Na^+^ [M+Na]^+^: 211.0477; found: 211.0478.

**1H-naphtho[2,3-d]imidazole-4,9-dione (2)**

To synthesize the titled compound, diamine **1** (1.6 g, 8.5 mmol, 1.0 equiv.) was suspended in 15ml formic acid and stirred at 110 ^o^C for overnight. After completion of the reaction, distilled water (20 mL) was added resulting in a yellow precipitate. To neutralize the excess formic acid, ammonium acetate was added until pH 7 is reached. The resulting brown solution was filtered and the precipitate was washed with distilled water. The desired product was obtained as a muddy yellow solid (1.53 g, 0.89 mmol, 89 %).

**^1^H NMR** (400 MHz, DMSO) 𝛿 (ppm): δ 8.2 (s, 1H), 8.1 (dt, *J* = 7.5, 3.8 Hz, 2H), 7.8 (dt, *J* = 5.7, 3.6 Hz, 2H).

**^13^C NMR** (101 MHz, DMSO) 𝛿 (ppm): δ 177.3, 144.3, 139.3, 133.7, 133.0, 126.2.

**HRMS (ESI+):** m/z calculated for C_11_H_6_N_2_ O_2_Na^+^ [M+Na]^+^: 221.0319; found: 221.0321.

**1,3-dimethyl-4,9-dioxo-4,9-dihydro-1H-naphtho[2,3-d]imidazole-3-ium tetrafluroborate (3)**

To synthesize the titled compound **3,** the imidazole **2** (735 mg, 3.7 mmol, 1.0 equiv.) was taken in Schleck flask then toluene (28 mL) and sodium hydride (60 % in mineral oil) (252.4 mg, 6.31 mmol, 1.7 equiv.) was added. After stirring for 4 h at room temperature, methyl iodide (0.92 mL, 14.8 mmol, 4.0 equiv.) in 28 mL DMF was slowly added to the reaction mixture. Then it was stirred overnight at 110 ^o^C. After cooling the reaction mixture to room temperature, diethyl ether was added to result an off-white precipitate. The precipitate was filtered and vacuum dried before being dissolved in 200 mL distilled water and then NaBF_4_ (446 mg, 4 mmol, 1.1 equiv.) was added. The reaction was stirred for 4-5 h until red precipitate was formed. The precipitate was filtered and washed with water. The final compound was obtained as red solid after vacuum drying. (198 mg, 0.63 mmol, 17 %)

**^1^H NMR** (400 MHz, DMSO) 𝛿 (ppm): δ 9.6 (s, 1H), 8.3-8.2 (m, 2H), 8.1 – 8.0 (m, 2H), 4.2 (s, 6H).

**^13^C NMR** (101 MHz, DMSO) 𝛿 (ppm): δ 174.9, 143.8, 135.3, 131.8, 130.8, 126.9, 36.1.

**HRMS (ESI+):** m/z calculated for C_13_H_11_N_2_O_2_^+^ [M]^+^: 227.0814; found: 227.0815.

**1,3-dimethyl-4,9-dioxo-4,9-dihydro-1H-naphtho[2,3-d]imidazol-3-ium acetate (4)**

Following a literature procedure [^1^], a fritted column for silica chromatography was filled with ion exchange resin (Amberlyst A26-OH, 20.0 g). NH_4_CH_3_CO_2_ (6.00 g) was dissolved in water (300 mL) and the column was flushed with aq. NH_4_CH_3_CO_2_ solution until the pH of the eluted solvent was about 9. Afterwards, the column was flushed with MeOH (100 mL, 2 times), while after the last cycle, the column was gently rotated to remove air bubbles from the resin material and ensure appropriate packing. After the settlement of all resin material, the solvent was eluted to the level of resin in the column. The corresponding imidazolium tetrafluoroborate salt (100 mg, 0.318 mmol, 1.0 eq.) was dissolved in methanol (5 mL) and added onto the resin. The solvent was eluted to let the solution penetrate the resin material, then the column was refilled with MeOH (100 mL). The solvent was collected in reaction tube fractions and checked for the elution of the imidazolium salt via TLC plate staining under UV light (254 nm) exposure. The positive fractions were combined and the solvent was removed by rotary evaporation (temperature should not exceed 30 °C). Drying of the remaining residue in an oil pump vacuum (< 1 mbar) lead to crystallization of an orange solid (40.0 mg, 0.140 mmol, 44%). For regeneration, the column was washed with aq. KOH solution (4.00 g in 200 mL H2O) until the pH was 14.

**^1^H NMR** (400 MHz, H_2_O) 𝛿 (ppm): δ 8.2– 8.16 (m, 2H), 7.9– 7.8 (m, 2H), 4.25 (s, 6H), 1.91 (s, 3H).

**^13^C NMR** (101 MHz, DMSO) 𝛿 (ppm): δ 180.1, 176.0, 135.5, 131.7, 131.3, 127.3, 36.0, 22.4.

**HRMS (ESI+):** m/z calculated for C_13_H_11_N_2_O_2_^+^ [M]^+^: 227.0814; found: 227.0815.

**Direct inlet EIMS:** m/z calculated for C_13_H_10_N_2_O_2_^·+^= [M]^·+^: 226.1, at 70 (±20) °C found: [M]^·+^: 226.1

## **1.3 NMR Spectra**

#### **1.3.1 1,3-dimethyl-4,9-dioxo-4,9-dihydro-1H-naphtho[2,3-d]imidazole-3-ium tetrafluroborate**

^1^H spectrum (400 MHz, DMSO-d6)

^13^C spectrum (100 MHz, DMSO-d6)

#### **1.3.2 1,3-dimethyl-4,9-dioxo-4,9-dihydro-1H-naphtho[2,3-d]imidazol-3-ium acetate**

## **1.4 Direct-inlet spectra of Rex(H)-CH_3_CO_2_ precursor**

**
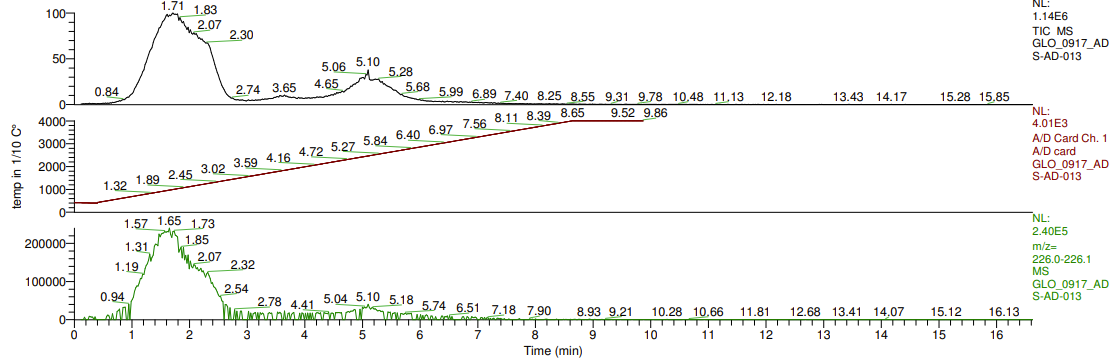
**

**
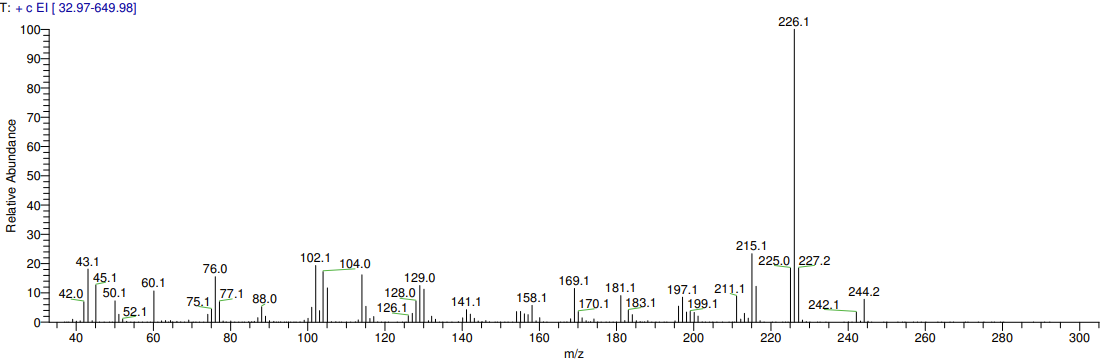
**

# **2. Self-assembled monolayer formation on Au^TS^ with NHC**

## **2.1 Au^TS^ surface preparation**

The bottom electrodes were prepared following previously reported procedure.[^2^] In short, 100 nm Au were deposited on clean Si (100) wafers using a thermal evaporator. Clean glass slides were then glued onto the Au surface with thermal glue (EPOTEK 353ND) and cured at 120 °C for 1 hour. The whole stack (metal, glue and support) was lifted from the wafer (which was the template) to expose the Au surface that had been in contact with the template and immediately immersed in the corresponding ethanolic carbene solution to minimize contamination of the metal surface by the ambient environment.

## **2.2 Modification with NHCs**

NHC-SAMs were formed using a modified literature-known procedure.[^1–3^] The NHC-acetate adduct was used here as an NHC-precursor, imidazolium-acetate adducts are widely used in organocatalysis and are known to generate free-carbenes.[^4,5^] A stock solution of the NHC-acetate precursor was prepared by dissolving the salt in ethanol to achieve a 1mM concentration, then 3 mL of this solution was transferred into a screw-capped glass vial.

The Au^TS^ surface was added immediately after template stripping without any washing. The vial was purged with argon then closed and placed in a temperature-controlled metal block and kept for 24 h. After the adsorption time, the coated Au^TS^ surfaces was removed from the solution and rinsed with ethanol (~ 10 sec. under a constant flow from a clean beaker). After drying under an argon flow (~ 30 sec.), the surface was placed in a new glass vial and back-filled with argon.

# **3. Surface characterization**

## **3.1 X-ray photoelectron spectroscopy (XPS) measurements**

**Table S1**: XPS measurement parameters.

| **XPS** | **X-ray source** | **Spot and detection** | **Charge compensation** | **Pass Energy (eV)** | **Dwell time**  **(ms)** | **Step size (eV)** | **No. of Scans** |
| --- | --- | --- | --- | --- | --- | --- | --- |
| Survey | Al Kα, 12 kV filament | 100 µm (72W max.). 60° x-ray incidence angle, 0° take-off angle (rel. to surface normal) | Metallic clips on thin film connected to grounded sample stage. | 200 | 100 | 0.9 | 2 |
| Au4f |  |  |  | 50, 100 | 50, 100 | 0.05 | 2, 18 |
| N1s |  |  |  | 100 | 100 | 0.05 | 18 |
| C1s |  |  |  | 50 | 50 | 0.05 | 5 |
| O1s |  |  |  | 50 | 50 | 0.05 | 5 |

### **3.1.1 NHC identification**

XPS measurements were performed on Thermo Fisher Scientific K-Alpha instruments (Organisch-Chemisches Institut, Corrensstr. 36, 48149 Münster). All spectra were referenced to Au 4f_7/2_. The spectra were analyzed by the use of Avantage 5.9925 (Thermo Fisher Scientific). Data visualization was carried out with Origin Pro 2023. Characterization of NHC on Au^TS^ was performed by comparing N1s core spectra to literature values.[^1,3,6–8^] Au-bound NHC is assigned to a single peak with a binding energy ranging between 400.0 eV and 401.0 eV. To distinguish the N1s between the carbene and the corresponding imidazolium salt, we also measured N1s core spectra of the imidazolium-BF_4_ salt, which was drop-casted onto a freshly prepared Au^TS^ surface under identical conditions mentioned in Table S1. In this case a peak at 402.1 eV was observed which is assigned to the physiosorbed imidazolium-salt, this value is also consistent with other literature reports.[^9^] We also collected N1s core spectra for standard surface but without washing with ethanol and in this case along with the carbene component, the physiosorbed imidazolium-salt is also present, matching the N1s signal observed in the drop-casted imidazolium-BF_4_ salt sample.


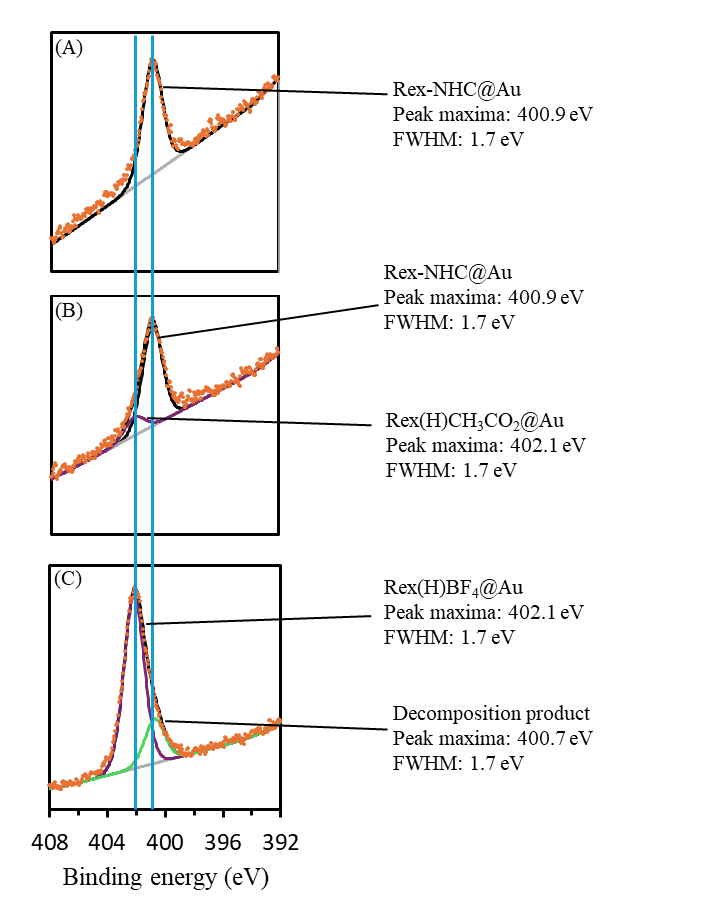


**Figure S2**: N1s control experiments, (A) Rex-NHC@Au, 24h sample from 1mM ethanolic solution at room temperature (standard deposition conditions). (B) Rex-NHC@Au prepared using standard deposition techniques but without washing after removal from the solvent. (C) Rex(H)BF4 salt was drop-casted on an Au surface.

For fitting, we assumed that the peak broadening and peak shape remained constant for identical measurement parameters. In this study, we used a peak full width at half maximum (FWHM) of 1.7 eV and GL30 function, for which we yielded the reported fit of NHC@Au (Figure S2, (A)). Similarly, we also obtained the fittings for the unwashed sample (Figure S2, (B)) and the imidazolium-salt drop-casted sample (Figure S2, (C)). Based on the assumption of FWHM remains constant, a clear distinction can be made between the component for the carbene bound to Au and the physisorbed imidazolium salt. An additional peak was also observed in case of the drop-casted imidazolium BF_4_-salt sample where the N1s core signal did not match the carbene or the imidazolium salt, and is most likely associated with a decomposition product as reported previously.[^6,10^]

### **3.1.2 Experimental estimation of surface coverage**

For estimating the surface coverage experimentally, we assumed that the NHC monolayer follows a single-layer adsorption process. Here, we assume that the N1s photoelectrons are not attenuation in the monolayer, so the XPS N1s signal intensity ($I_{N1s}$) is expected to be proportional to the amount of nitrogen ($N_{Nitrogen}$) and hence the amount of NHC ($N_{NHC}$) present at the surface:

$$I_{N1s} \propto N_{Nitrogen} \propto N_{NHC} (1)$$

Marder and coworkers also reported a method of experimental determination of surface coverage.[^11^] They estimated the amount of NHC molecules per unit area by corelating the XPS Au4f signal intensity ($I_{Au4f}$) with the XPS N1s signal intensity (I_N1s_) as shown in equation (2). Here they ignored the attenuation of Au4f signal and N1s signal by passing through the monolayer of NHC molecules.

$$\frac{N_{NHC}}{A}=\frac{1}{2}\frac{N_{Nitrogen}}{A}=\frac{1}{2}\frac{I_{N1s}}{I_{Au4f}}\frac{N_{Au}}{V}\lambda_{Au4f,Au}^{IMFP} (2)$$

in which the signal intensities $I_{N1s}$ and $I_{Au4f}$were measured under identical instrument settings (see Table S1) and corrected with the instrument- and element-specific sensitivity factor. $\lambda_{Au4f,Au}^{IMFP}$ is the inelastic mean free path (IMFP) of a Au4f photoelectron in the Au layer and $\frac{N_{Au}}{V}$ is the amount of Au atoms per volume.

Building on these equations, our group also recently reported another method of surface coverage, where we replaced the inelastic mean free path by effective attenuation lengths (EALs), allowing us to account for elastic photoelectron scattering. We also considered the attenuation of Au4f signal by a NHC monolayer of thickness d by:

$\frac{I_{Au4f}}{A}=\frac{I_{Au4f}^{\infty}}{A}e^{{-d}/{\lambda_{Au4f,NHC}^{EAL}}}$(3)

Where $I_{Au4f}^{\infty}$ is the Au4f signal intensity of a sputter clean Au surface and this is given by:

$\frac{I_{Au4f}^{\infty}}{A}=k \lambda_{Au4f,Au}^{EAL}\frac{N_{Au}}{V}$ (4)

As both the attenuated (with monolayer) and non-attenuated (sputter-cleaned surface) Au4f signal intensities were measured, we could estimate the thickness d of the monolayer at saturation by equation 3.

Further if we also considered the attenuation of N1s signal by passing through the NHC monolayer.

$$I_{N1s}\left( t \right)=k N_{Nitrogen}\left( t \right)e^{\frac{-t}{\lambda_{N1s,NHC}^{EAL}}} (5)$$

Where, $I_{N1s}\left( t \right)$ is the contribution to the total N intensity from depth t, $N_{Nitrogen}(t)$ are the number of nitrogen atoms at depth t and $\lambda_{N1s,NHC}^{EAL}$ is the effective attenuation length of nitrogen photoelectrons passing through the NHC monolayer.

In this case, to include the contribution from all the depths above the gold surface, we have to integrate from 0 to d, where d is the monolayer thickness, we experimentally deduced from equation 3. Then the $\frac{I_{N1s}}{A}$ will be given by equation 6.

$$\frac{I_{N1s}}{A}=\frac{k}{d} \frac{N_{Nitrogen}}{A} \lambda_{N1s,NHC}^{EAL} [1-e^{\frac{-d}{\lambda_{N1s,NHC}^{EAL}}}] (6)$$

Now, combining equation 3, 4 and 6, we can deduce the equation for $\frac{N_{NHC}}{A}$ as follows:

$$\frac{N_{NHC}}{A}=\frac{1}{2} \frac{I_{N1s}}{I_{Au4f}} \frac{N_{Au}}{V} d \frac{\lambda_{Au4f,Au}^{EAL}}{\lambda_{N1s,NHC}^{EAL}} \frac{e^{\frac{-d}{\lambda_{Au4f,NHC}^{EAL}}}}{[1-e^{\frac{-d}{\lambda_{N1s,NHC}^{EAL}}}]} (7)$$

Table S2: Estimation of surface coverage through XPS

| Equation used | $\frac{N_{Au}}{V}$ | $\lambda_{Au4f,Au}^{IMFP}$ | $\lambda_{Au4f,Au}^{EAL}$ | $\lambda_{N1s,NHC}^{EAL}$ | d | $\frac{N_{NHC}}{A}$ |
| --- | --- | --- | --- | --- | --- | --- |
| Eq 2 | 58.94 nm^-3^ | 2.02 nm (IMFP) | - | - | - | 3.95 × 10^14^ cm^-2^ |
| Eq 6 |  | 1.62 nm (EAL) | 3.89 nm | 3.09 nm | 0.8 nm | 2.91 × 10^14^ cm^-2^ |

The calculated values above are obtained using $I_{N1s}$ and $I_{Au4f}$ which are corrected for instrument- and element- specific sensitivity factors. Our estimation of surface coverage using eq. 2 is significantly higher than using eq. 6 where we account for signal attenuation even for the NHC monolayer. However, Surface coverage obtained from this method can’t account for the effects of orientational changes on photoelectron attenuation and hence is referred as an “estimation”. Notably coverage estimation obtained from CV studies (2.32× 10^14^ cm^-2^) are quite similar to our estimation using eq. 6, further supporting the reliability of our calculations.

### **3.1.3 XPS spectra of Rex-NHC@Au**


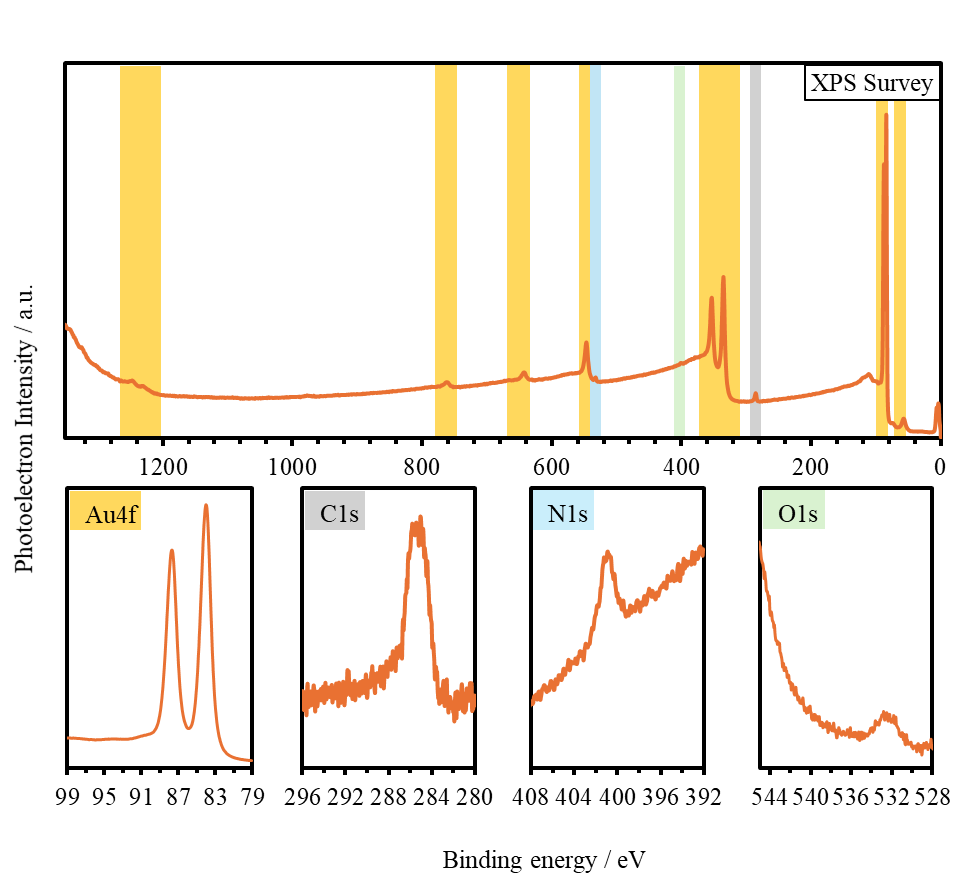


**Figure S3**: XPS spectra for Rex-NHC@Au under standard deposition conditions, (24h, 1mM ethanolic solution of imidazolium-acetate precursor), the measurement parameters are summarized in Table S1.

## **3.2 IRRAS**

A variable angle reflection accessory (Bruker A513/Q) was used and set to 75°. The resolution was set to 2 cm^−1^, and the aperture was set to 1.5 mm. A set of 10 times 200 scans were collected for each p- and s-polarization. A plain Au layer served as a baseline. The final spectra were corrected for atmospheric water and the baseline was corrected using an endpoint straight line.

We measured the Rex-NHC salt in a KCl pellet using transmission FT-IR and compared it to the Rex-NHC grafted on template-stripped gold (Au^TS^) using FT-IR reflection absorption spectroscopy (Figure 5a top two panels). In the following we highlight the bands most relevant to our discussion. We assigned the band at 1678 cm^-1^ to the carbonyl stretching of the quinone moetiy, the band at 1259 cm^-1^ to the CH_3_ bending mode bound to the N, the band at 940 cm^-1^ to the in plane ring stretching and the band at 716 cm^-1^ to the out of plane ring mode (oop) of the Rex-NHC backbone. The assignment of these bands was done comparing previous literature confirmed with DFT calculation via ORCA software (see DFT section).[^11–14^] The C=O stretching vibration, the CH₃ bending mode, and the out-of-plane oop ring deformation can be associated with distinct orthogonal directions relative to the gold surface: the C=O mode aligns along the x-axis (parallel to the surface), the CH₃ bend corresponds to the y-axis (also in-plane but orthogonal to the C=O direction), and the oop ring mode projects along the z-axis (normal to the surface). We used these three bands as orientation reporters within Debe’s intensity-ratio method,[^14^] including partial orientational disorder (in our case caused by different SAM phase domains) via the ordered fraction *f* (equivalently α=(1-*f*)/(3*f*)). We propagated the experimental uncertainty in the band intensities and marginalized the tilt angle *θ* over the data-constraining interval *f* = 0.85–1.00 (for *f* ˂ 0.85, *θ* becomes highly noise-sensitive). This yields an ensemble estimate of *θ* = 17 ± 6°, consistent with a predominantly upright monolayer and a value reported by Cyganik and co-workers.[^15^] As a limiting reference, enforcing *f* = 1 gives *θ* =21 ± 4°.

## **3.3 Atomic force microscopy**

We have characterized the surface topography using atomic force microscopy (AFM). Figure S4a shows the AFM image along with a height profile (Figure S4b) taken from this images as indicated with the dashed white line. The root-mean squared (rms) surface roughness of 1.4 ± 0.3 nm recorded over an area of 5×5 μm^2^ is consistent with a homogeneous SAM-covered polycrystalline Au surface. Our findings are comparable to the rms surface roughness reported by others for similar NHC-derived monolayers on gold surfaces.[^16,17^]

##
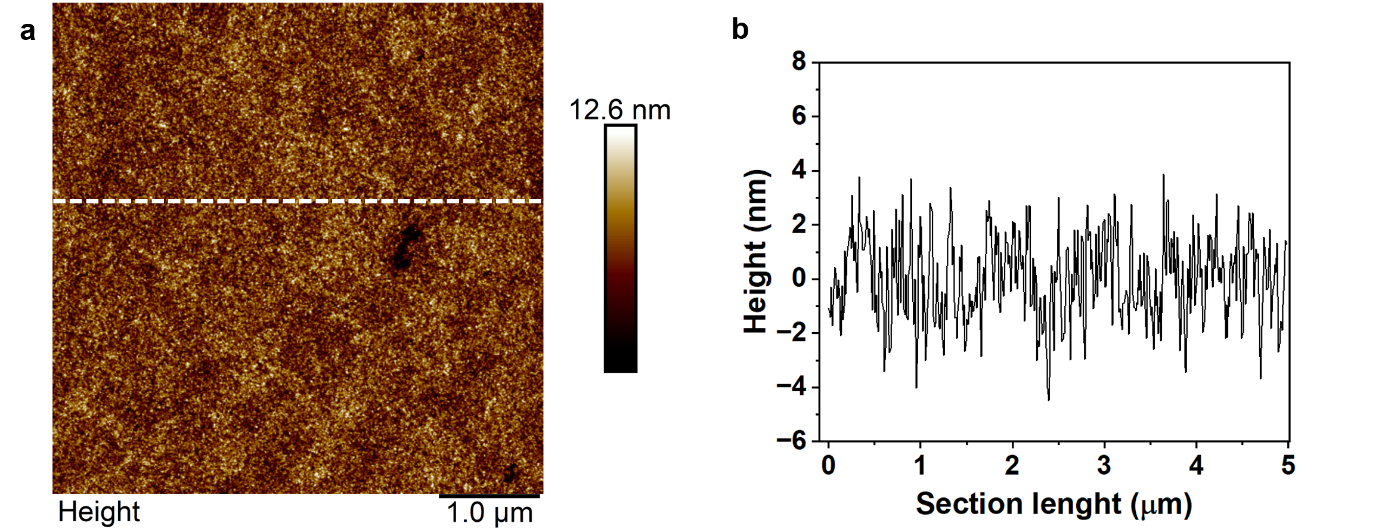


**Figure S4** a) AFM images 5⨯5 μm^2^  of Rex SAMs and b) line scans of 5 micron image.

## **3.4** **Sum-frequency generation (SFG) spectroscopy**

Vibrational SFG spectroscopy was done with a homebuilt SFG spectrometer that was described in detail elsewhere.[^18^] In brief, we have used a Spectra Physics Soltice chirped-pulse amplifier system that emits laser pulses at 798 nm with about 7 mJ pulse energy, 23 nm bandwidth and 70 fs pulse duration. These pules were slit in two beam paths with a about equal pulse energy. This is then used to pump a Light Conversion optical parametric amplifier (TOPAS prime) with subsequent nonlinear difference frequency generation (NDFG), where as the second pulse is filtered with an etalon to yield narrowband pulses at 804.1 nm with a bandwidth of <5 cm^-1^. SFG spectra were collected in a reflecting geometry with the mid IR pulse (generated from the TOPAS and NDFG units) and the narrowband pulses having angles of incidence of 60° and 55°, respectively. All spectra were collected with the mid IR, narrowband and sum-frequency beam being p-polarized. The pulse energies used in the experiments were 10 µJ and 17 µJ for the mid IR pulse with a bandwith of >300 cm^-1^ and the narrowband pulse. In order to suppress the nonresonant contribution of the Au to some extend we used the method described by Lagutchev et al. [^19^] and delayed the narrowband pulse by ~50 fs. Detection of SFG photons was done by using a Andor (Kymera) spectrograph with a 1200 lines/mm grating and a Andor (Newton) EMCCD.

## **3.5 Electrochemistry**

The cyclic voltammetry measurements of the Rex SAMs were conducted with an Autolab PGSTAT302T setup equipped with NOVA 1.10 software. In the measurements, a Pt mesh worked as the counter electrode, an Ag/AgCl electrode was used as the reference electrode, the Au substrate coated with SAM operated as the working electrode with geometric area of 0.282 cm^2^, and pH 2 NaClO_4_ aqueous solution (0.1 M) was used as the electrolyte. We recorded the results between -0.5 V and 0.25 V with various scan rates from 0.02 V/s to 1 V/s. The Rex SAMs on the Au were derived from compound 4 and measured with cyclic voltammetry (Figure S5a). The CVs at all scan rates show quasi-reversible peaks, which is consistent with the typical 2e^-^, 2H^+^ charge transfer reaction of quinones (Figure S5b).[^20^] The cyclic voltammograms show one quasi reversible redox wave similar to previously reported CV quinones SAMs in aqueous electrolyte. The surface coverage of the Rex SAM (Γ_Rex_) was calculated with equation S1. The value of Γ is (3.86 ± 0.03) × 10^-10^ mol/cm^2^, which was calculated at a scan rate of 0.5 V/s by integrating the anodic peak and taking electrons transferred number n = 2.[^2,21^]

$\Gamma_{\mathrm{Rex}}=\frac{Q_{tot}}{vnFA}$ (S1)

where Γ is the surface coverage of SAM (mol/cm^2^), Q_tot_ is the total charge integrated from the redox wave, n is the number of electrons transferred in the redox reaction, F is the Faraday constant (96485 C/mol), ν is the scan rate (V/s) and A is the contact area of the working electrode with the electrolyte (0.282 cm^2^).

**
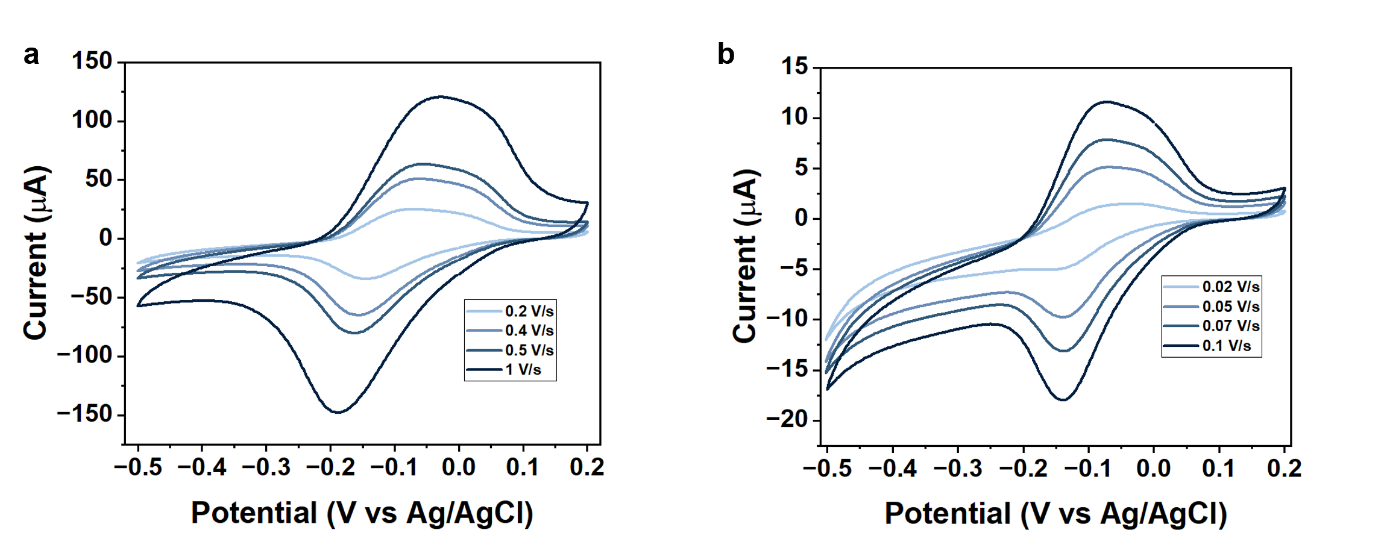
**

**Figure S5.** Cyclic voltammograms of Rex SAM on Au in 0.1 M aqueous NaClO_4_ at pH 2 recorded with scan rates in the ranges of 0.02-0.1 V/s (a) and 0.2-1 V/s (b).

The energy levels of the Rex SAM were also studied using CV measurements.[^2^] The LUMO level (E_LUMO_) of the Rex SAM was accounted with the equation S2, with the value about -4.59 eV. To further probe the energy gap (E_gap_) between the HOMO and LUMO levels obtained from the UV measurement, the HOMO level (E_HOMO_) was also calculated with the equation E_LUMO_ = E_HOMO_ + E_gap_.

*E*_LUMO_ = *E*_abs, NHE_ - e*E*_1/2,NHE_ (S2)

wherein, E_abs_,_NHE_, equals to -4.5 eV, is the absolute potential energy of the normal hydrogen electrode, e is the charge of one electron (1.602×10^-19^ C), and E_1/2,NHE_ is the formal half-wave potential versus normal hydrogen electrode (NHE). The absolute potential energy of Ag/AgCl is +0.197 V vs. NHE**.** The absolute potential energy of Ag/AgCl is +0.197 V vs. NHE**.** The HOMO level was found to be about -7.81 ± 0.01 eV.

## **
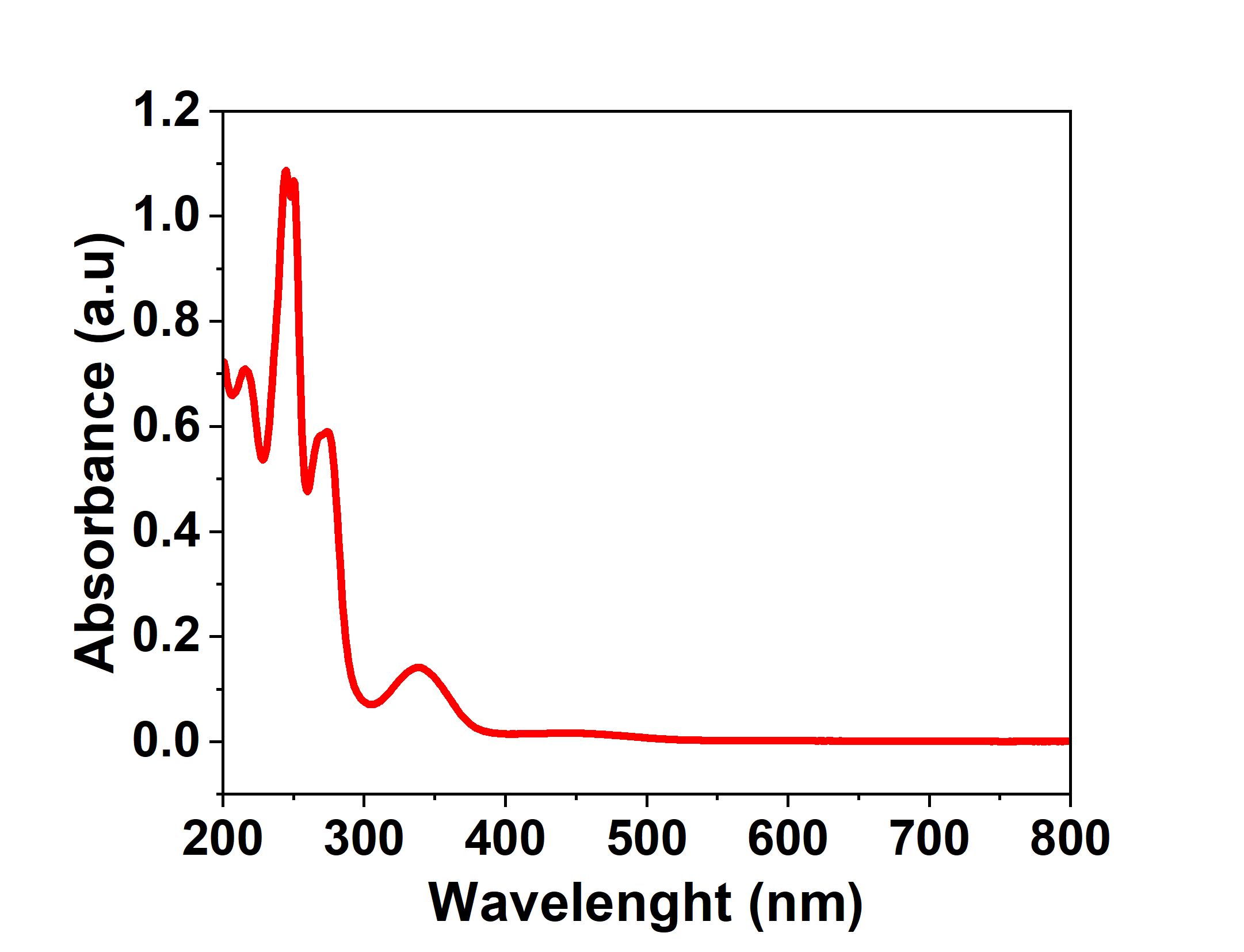
3.6 Uv-vis spectroscopy**

**Figure S6.** UV-vis spectra of Rex-NHC-acetate in EtOH.

The UV-vis spectra of Rex-NHC acetate indicate light absorption in the range of 380-300 nm, and high absorption peaks with the main peak at 245 nm and three shoulder peaks around 273, 250 and 216 nm, which are similar to this spectrum. The main peak is assigned to the π–π* transitions associated with the napthoquinone core and the peak at 336nm is associated to the n- π* transition of the C=O.[^22^] We used the onset of the absorption peak to extract the optical HOMO-LUMO energy gap of 3.22 eV.

**Table S3**. Summary of surface characterization of Rex molecules on Au

| *Γ*^c^  (10^-10^ mol/cm^2^) | *E*_HOMO_  (mV) | FWHM  (mV) | *E*_pa_  (mV) | *E*_pc_  (mV) | Δ*E*_p_  (mV) |
| --- | --- | --- | --- | --- | --- |
| 3.86±0.03 | -7.81±0.03 | 164 ± 1 | -163 ± 1 | -60 ± 1 | 66 ± 1 |

# **4. EGaIn J-V measurements**

The statistical analysis to obtain the log-average J(V) curves has been described in detail elsewhere.[^2,23,24^] Briefly, we plotted histograms of |*J*| values on a log scale, assuming a log-normal distribution to which we fitted Gaussians to obtain log-mean value of |*J*|, log_10_|*J*|_G_, and the log-standard deviations (*σ*_log_) which we used to plot the log-average *J*(V) curves. Figure S7 shows two examples of these histograms along with Gaussian fits and log-average *J*(V) curves where the error bars represent *σ*_log_ for junction measured at relative humidity of 40% and <5%.[^21,25^] We recorded 10 scans per junction across 45 junctions from 5 samples.

**Table S4**. Summary of EGaIn characterization of Rex molecules on Au

| **No. of junctions** | **No. of shorts or unstable junctions** | **No of traces** | **Yield** | **Log_10_ R_on/off_** |
| --- | --- | --- | --- | --- |
| 45 | 10 | 450 | 90% | 1.91±0.3 |

**
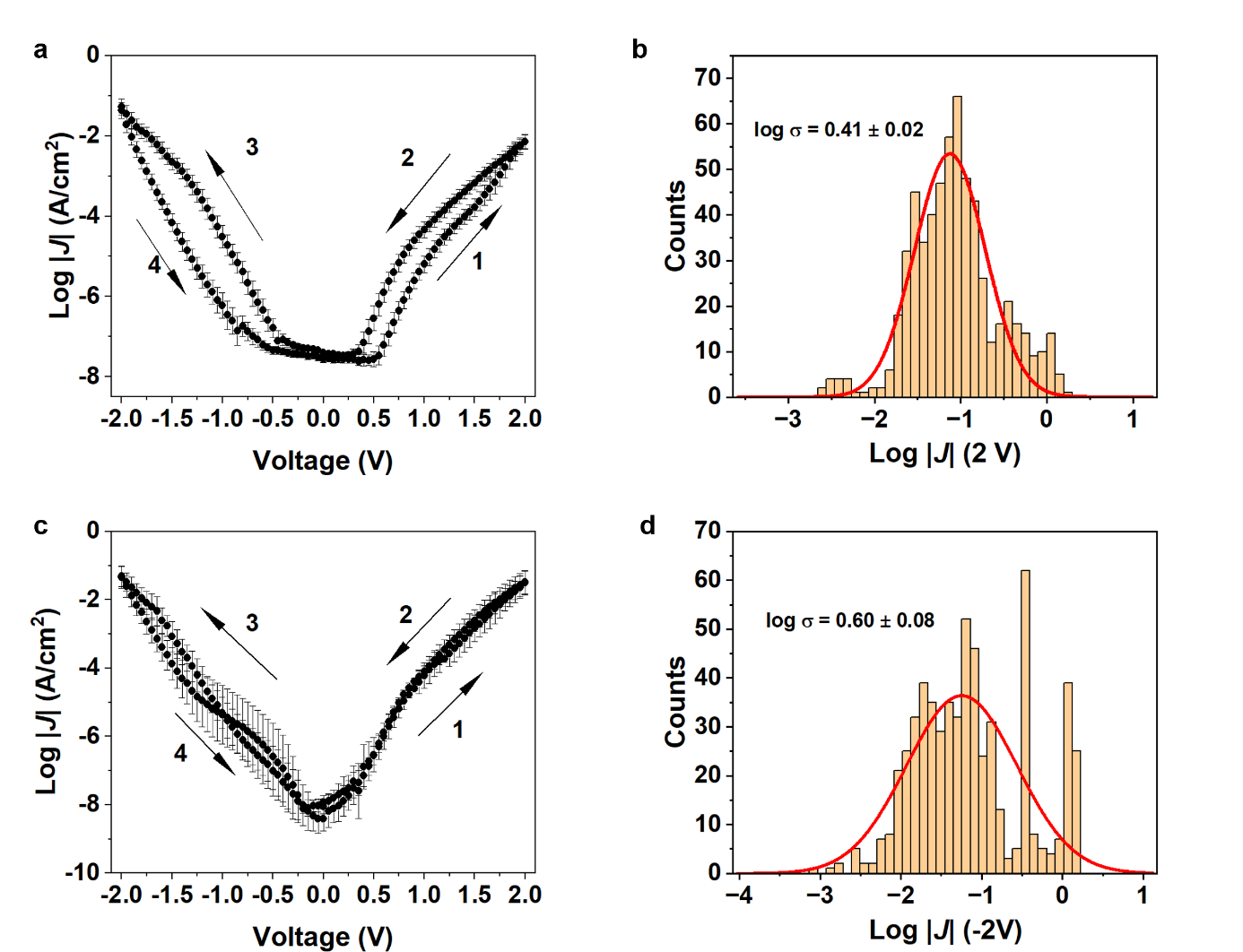
**

**Figure S7**. J-V curves Rex SAMs (a) and relative histogram of log standard deviation at ± 2V (b) J-V curves recorded at 1% relative humidity (c) and relative histogram of log standard deviation at -2V (d)

## **4.1 Voltage breakdown measurements, aging, retention and endurance**

The breakdown voltage was determined as follows. First, a stable junction was formed with a SAM and its *J*(V) characteristic was measured in the bias window of ±2 V to verify that the *J*(V) curve fell within one σ_log_ of the Gaussian log-mean *J*(V) curve. Next, we increased the bias in either positive or negative direction starting from 0 to +5.0 or −5.0 V till the junction broke down abruptly characterized by a sharp increase of *J* of several orders of magnitude. This experiment was repeated for 40-50 times and the *V*_bd_ values were plotted in histograms to which a Gaussian was fitted to determine the Gaussian average value of *V*_bd_, and the standard deviation. These SAMs were stored in ambient conditions for 2 months and showed stable on/off ratio over that time period (Figure S8c).

Figure S8d shows the write–read–erase–read (WRER) voltage sequence (write = 10 s, erase = 5 s, read = 3 s) and the corresponding current response, while Figure S8e shows the ON state after write (Ron, black) and the OFF state after erase (Roff, red). After an initial conditioning period (~60 cycles), the junctions reproducibly switch between on and off states with an on/off ratio of approximately one order of magnitude during WRER cycling. Figure S8f shows the retention test of the junction in the on and off states over 1500 s. During the first ~200 s, the ON state shows a clear time-dependent decay, whereas the off state remains comparatively stable; consequently, the separation between the two states decreases with time and then stabilizes at ~1.5 orders of magnitude.[^26,27^]


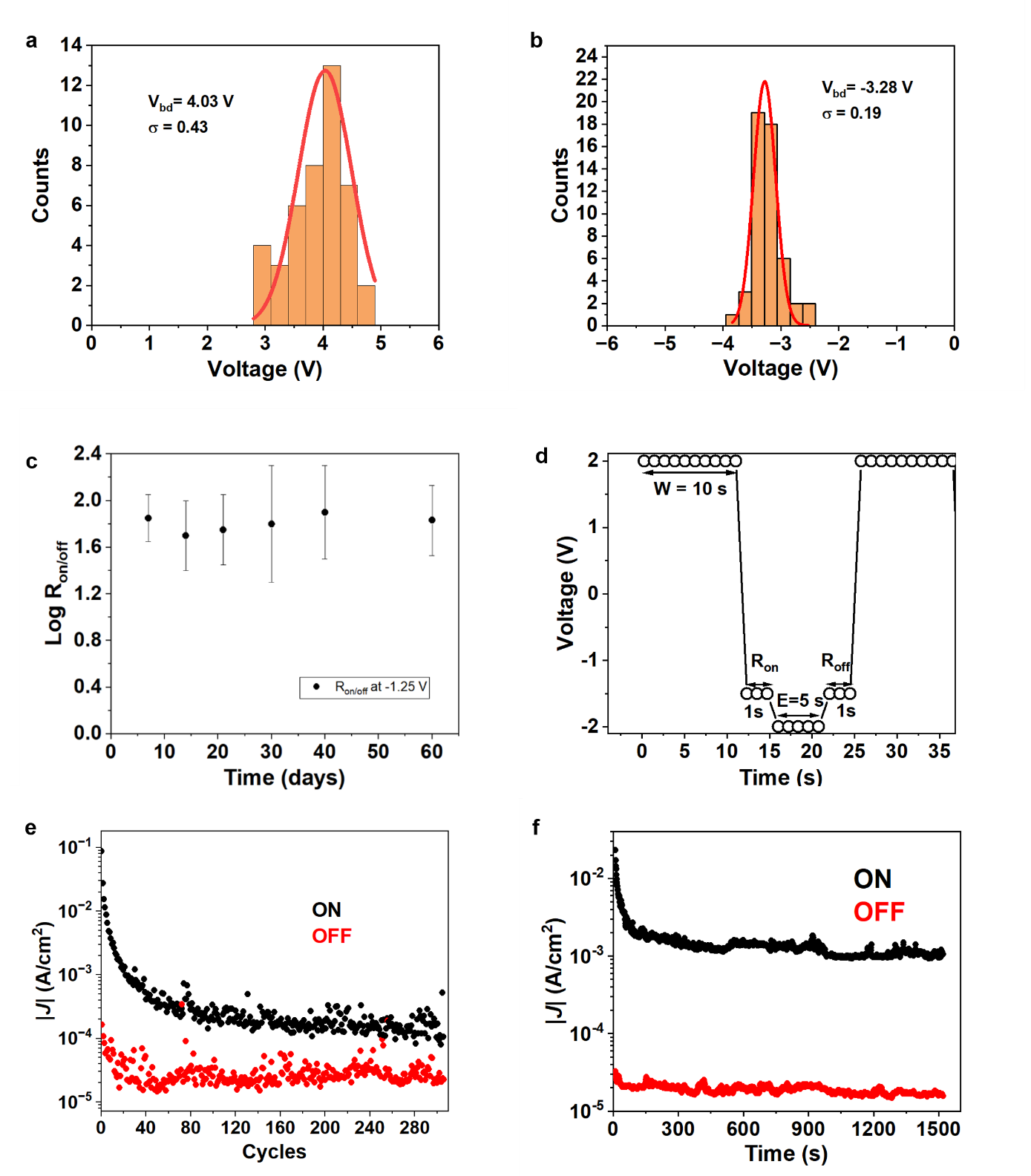


**Figure S8**. Histogram of the breakdown voltages at positive (a) and negative (b) along with Gaussian fits to these histograms. (c) Stability of log (*R*) at -1.25 V for the Rex-NHC junctions measured over a period of 60 days. The error bars represent standard deviations from multiple measurement positions across the same sample. (d) the WRER pulse sequence**,** (e) the endurance over 300 WRER cycles (f) current retention in the on (black) and off (red) states over 1500 s.

# **5. Computational Details**

Transport characteristics were evaluated using a first-principles approach based on density functional theory (DFT) integrated with the non-equilibrium Green’s function (NEGF) formalism.[^28,29^] The Green’s functions $G(E)$ were computed using the Hamiltonian ($H$), and overlap matrix ($S$) of the device region, and self-energies of the left and right electrodes $\Sigma_{L/R}(E)$,

$$G\left( E \right)=\left[ S.E-H-\Sigma_{L}(E)-\Sigma_{R}(E) \right]^{-1}$$

The transmission function $T(E)$ is then calculated using the equation

$T\left( E \right)=Tr\{\Gamma_{R}G\left( E \right)\Gamma_{L}{G\left( E \right)}^{\dagger}\}$,

where the left/right electrode coupling $\Gamma_{L/R}$ is the imaginary part of the self-energies, $\Sigma_{L/R}$

$\Gamma_{L/R}=i (\Sigma_{L/R} -{\Sigma_{L/R}}^{\dagger})$.

Geometry optimizations were performed for both quinone (QN) and hydroquinone (HQN) forms of the N-heterocyclic carbene (NHC) molecules in the gas phase using the PBE-D i.e., Perdew-Burke-Ernzerhof exchange-correlation functional [^30^] along with Grimme’s dispersion corrections (DFT-D) [^31^] and Becke-Johnson damping, with double-ζ polarized (DZP) basis set. As EGaIn electrode does not exhibit a well-ordered structure,[^32^] the molecular junctions were modeled with Au electrodes, employing 3×3 face-centered cubic (FCC) Au(111) slabs as both left and right electrodes.[^33,34^] Au-adatoms were modelled at the left electrode to interact with the carbene carbon. Two different packing densities were considered by placing either one or two NHC molecules between the 3×3 Au electrodes, corresponding to Au–$n$NHC–Au junctions ($n = 1, 2$). In the high-density configuration ($n$ = 2), the adjacent NHC molecules lie within π-stacking interaction range. The full junction geometries were optimized using the PBE-D functional, with a DZP basis set for the NHC molecules and DZ basis set for gold atoms. All Au atoms except the adatoms were kept fixed at the experimental Au(111) lattice position. A $4\times4\times1$ Monkhorst-Pack k-point grid, a mesh cutoff of 300 Ry, and a force convergence criterion of $0.04$ eV/Å were used for structural optimizations. The optimized geometries are available at [<https://iochembd.chem.ku.dk/browse/review-collection/100/334/1936d6f16aee08599928311b>]. To model a heteroelectrode configuration mimicking the asymmetry of the EGaIn setup, the atoms of the right Au electrode were substituted with Al atoms, without further geometry optimization. This approximation is justified by the close match in lattice constants (Au: 4.08 Å; Al: 4.05 Å) and similar work functions (Al ≈ 4.24 eV; EGaIn ≈ 4.1-4.2 eV).[^28^]

For transport calculations, additional Au/Al layers were added. Transmission functions were computed using the same level of theory with a denser $7\times7\times1$ Monkhorst-Pack grid. Finite-bias transport simulations were performed in the voltage range from $-1.0$ V to $+1.0$ V using non-equilibrium self-consistent NEGF approach at the same level of theory as in zero bias. All electronic structure calculations were carried out using the **SIESTA** package [^28^] ,and transport properties were evaluated using the **TranSIESTA** module.[^29^]

**Table S5:** NHC localized orbital energies in eV of Au-2NHC-Au junction. Calculated at PBE-D level.

|  | **H-1** | **H** | **L** | **L+1** |
| --- | --- | --- | --- | --- |
| **Au-2NHC(HQN)-Au** | -2.60 | -2.10 | +0.03 | +0.09 |
| **Au-2NHC(QN)-Au** | -1.73 | -1.70 | +0.06 | +0.61 |

**Table S6:** Transmission values at Fermi energy $T(E_{F})$ of Au-2NHC-Au junction and the ratio of transmission of HQN and QN form calculated at various bias.

| **Bias (V)** | **Transmission at** $\boldsymbol{E}_{\boldsymbol{F}}$ | | **Ratio of** $\boldsymbol{T(}\boldsymbol{E}_{\boldsymbol{F}}\boldsymbol{)}$ |
| --- | --- | --- | --- |
|  | **HQN** | **QN** |  |
| -1.0 | 7.09 $\times{10}^{-2}$ | 1.31 $\times{10}^{-4}$ | 541.2 |
| -0.5 | 5.72 $\times{10}^{-3}$ | 7.58$\times{10}^{-5}$ | 75.5 |
| 0 | 9.20 $\times{10}^{-3}$ | 1.01 $\times{10}^{-2}$ | 1.1 |
| +0.5 | 4.99 $\times{10}^{-4}$ | 6.43 $\times{10}^{-5}$ | 7.8 |
| +1.0 | 1.71 $\times{10}^{-4}$ | 6.06 $\times{10}^{-5}$ | 2.8 |

**
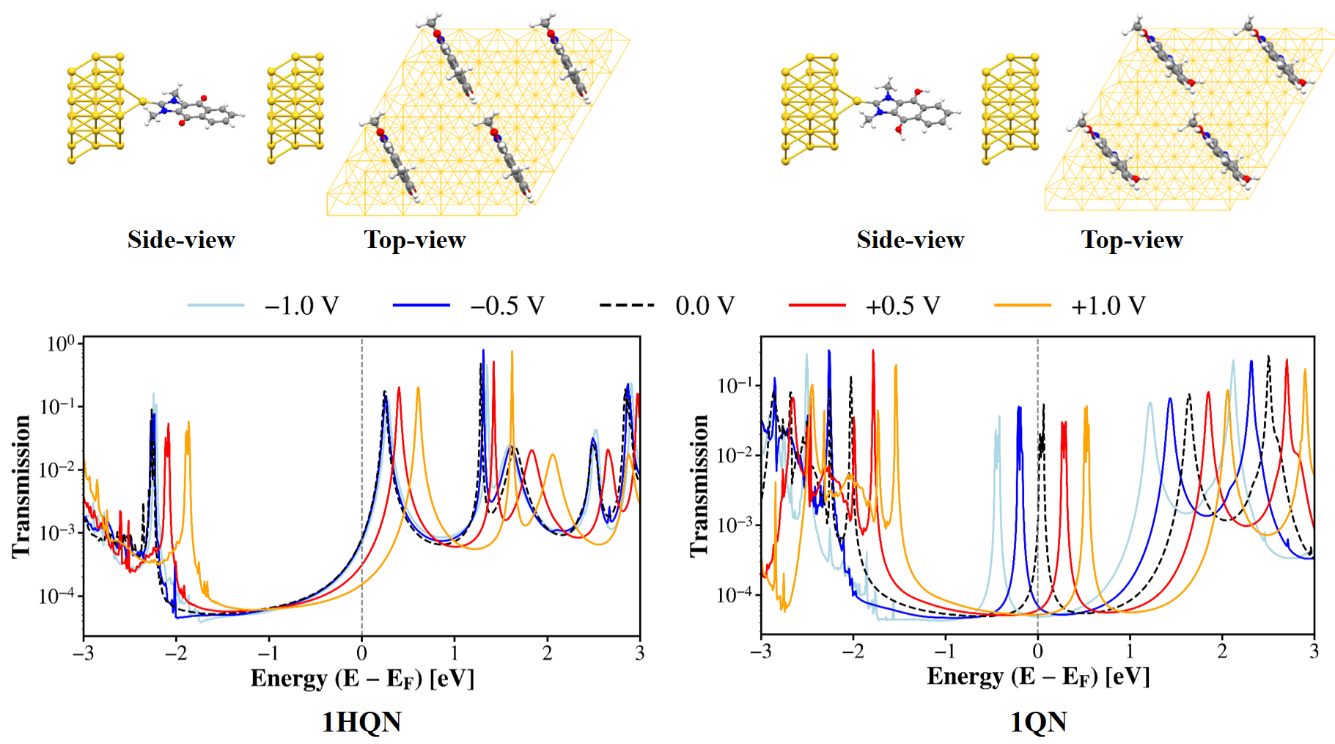
**

**Figure S9:** Side- and top-view of the optimized structures of Au-$1$NHC-Au junction for both HQN and QN form. H, C, N, O and Au are represented by white, grey, blue, red and yellow coloured balls, respectively. (Top panel) Calculated transmission function per molecule at $0.0$V, $\pm0.5$V and $\pm1.0$V for Au-$1$NHC-Au junction as a function of energy relative to the $E_{F}$ for both HQN and QN form. (Bottom panel)

**
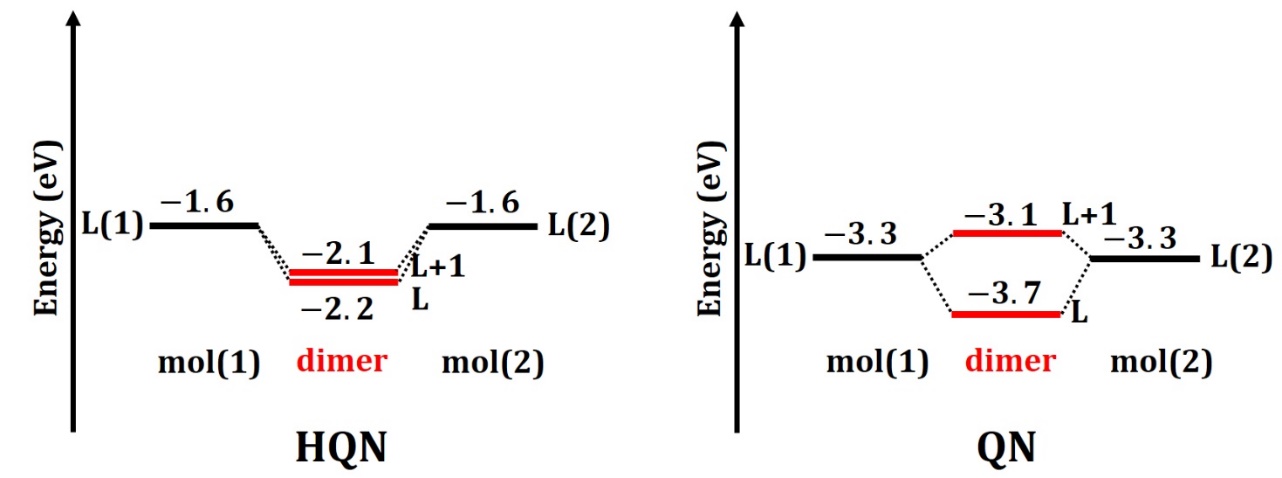
**

**Figure S10:** Schematic diagram of LUMO (L) energies of the monomeric unit and the dimeric NHCs extracted from the optimized junction geometry (by removing Au electrodes) for both HQN and QN form, calculated at PBE-D/DZP level.


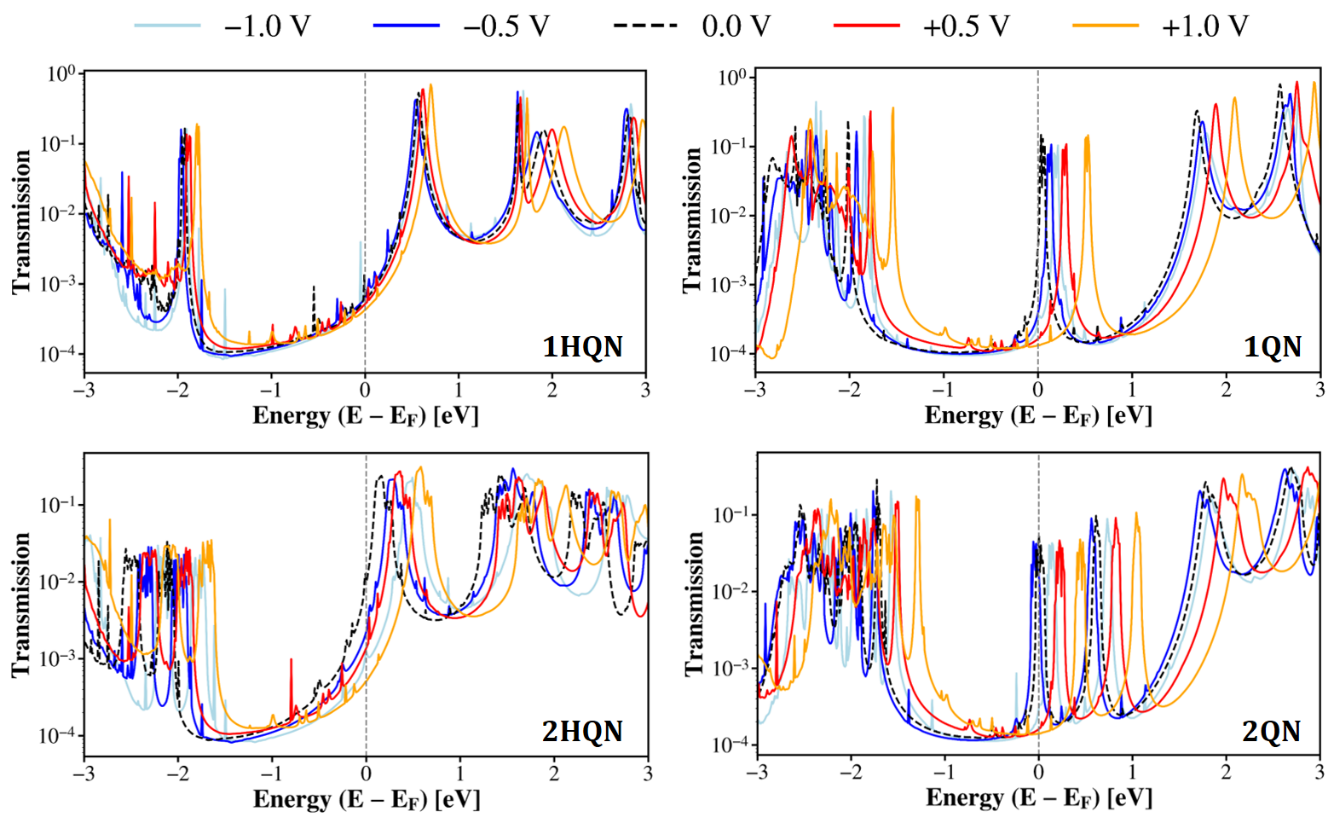


**Figure S11:** Calculated transmission function per molecule at $0.0$V, $\pm0.5$V and $\pm1.0$V for Au-$n$NHC-Al ($n=1, 2$) junctions as a function of energy relative to $E_{F}$ for both HQN and QN form.

**Impact of hetero-electrode junction:** Considering that the experimental setup employs an EGaIn top electrode, which has a different work function than gold, the junction forms a heteroelectrode configuration. This asymmetry can significantly influence the alignment of the Fermi level relative to the molecular orbital (MO) energies and, consequently, how the bias window (i.e., $E_{F}\pm\frac{eV}{2}$) is applied. To examine the effect of the work function difference, we modelled a heteroelectrode junction by replacing right Au electrodes with Al, yielding an Au$-n$NHC$-$Al ($n=1, 2$) structure. Corresponding transmission plots are shown in Figure S11. Interestingly, in this heteroelectrode setup, the application of both positive and negative bias shifts the NHC-localized LUMO peaks toward higher energies (*i.e.,* to the right in the transmission plot). This shift leads to a reduction in transmission for both QN and HQN forms, similar to the trend observed in the symmetric Au$-n$NHC$-$Au junction at positive bias. However, due to the sharper and narrow nature of the QN transmission peak, the decrease in transmission is more pronounced for QN than for HQN. As a result, the difference in bias response between the two redox states persists, yielding high on/off ratios even under heteroelectrode conditions.

#
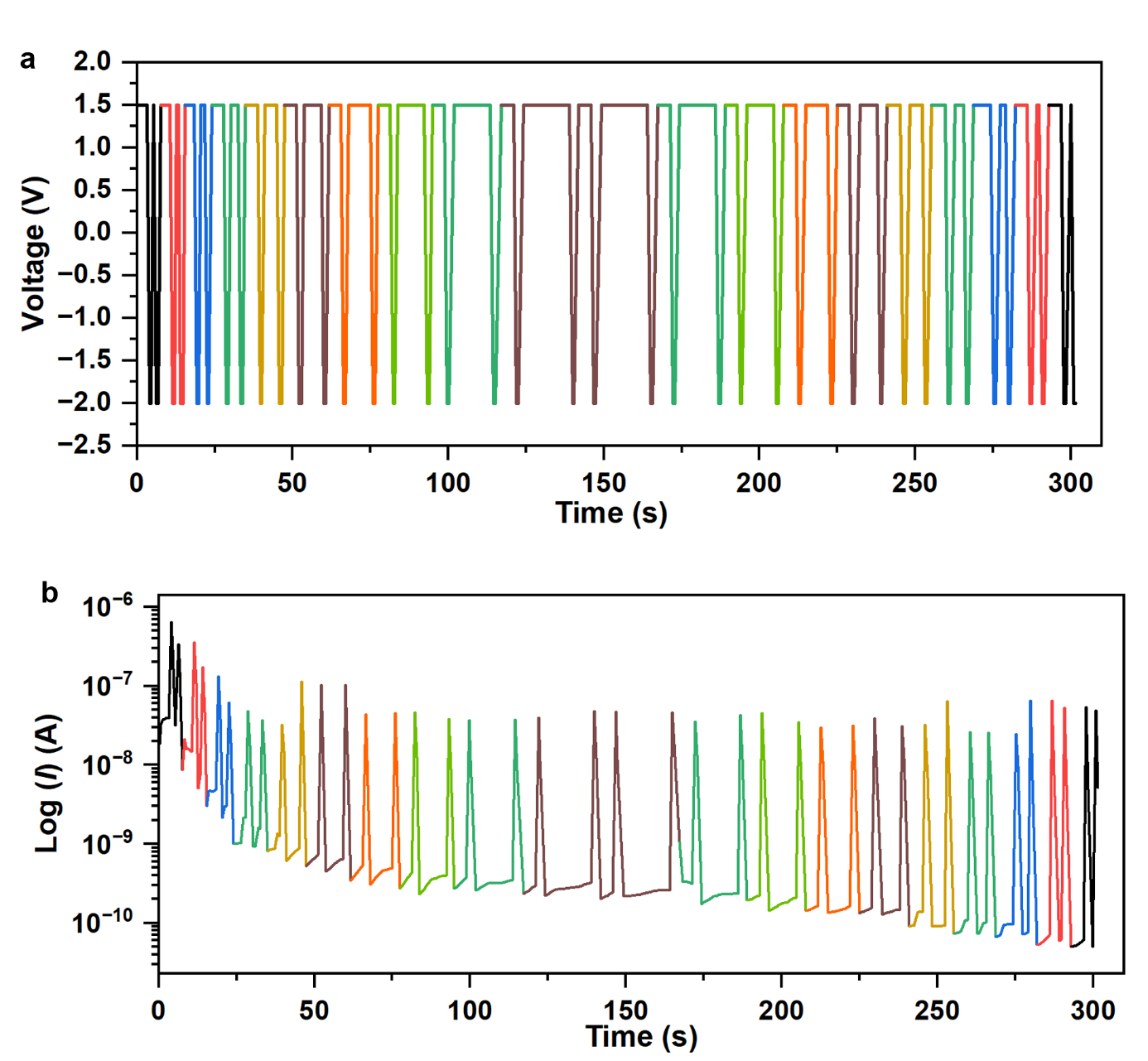
**6. Synaptic plasticity**

**Figure S12:** a) Voltage pulse sequence used in the measurement and logarithm current response (b) with color used to help distinguish sections of the sequence. Time *t* = 0 corresponds to the time the measurement was started, immediately after junction formation.

# **7. Pavlov learning**

Figure S13 provides the phase-specific equivalent circuits and the full pulse sequence used in the experiment. The conditioning protocol consists of four consecutive phases: (i) N1-only stimulation (“dog sees food”), (ii) N2-only stimulation before training (“dog hears bell”), (iii) simultaneous N1 and N2 stimulation (training), and (iv) N2-only stimulation after training (conditioned response). In the experiment, N1 alternates between −1 V and +1 V (2 s each, repeated 5 times), whereas N2 alternates between −2.1 V and −2.0 V (2 s each, repeated 5 times).The equivalent circuit for N1-only stimulation shown in Figure S13a (“Dog sees food”) is reduced to a form in where the input neuron N1, the synapse S1(R1) and the output neuron N3 are connected in series. The trainable synapse S2 which is not stimulated has a resistance which is orders of magnitude higher than R1, R3 and therefore S2 is not contributing to the current in N3. The current measured by the source meter acting as N1 is the same everywhere in this circuit. The source meter thus measured the current trough N3. The equivalent circuit for N2-only stimulation after training is shown in Figure S13b. The voltage applied by the source meter (N1) is 0V. Because both R1 and R3 are connected to 0V with respect to signal ground they are connected in parallel in the equivalent circuit. The current flowing in the trainable synapse S2 is equally divided over R1(10 kΩ) and R3(10 kΩ). The source meter (N1) thus indirectly measures the current trough N3.

**
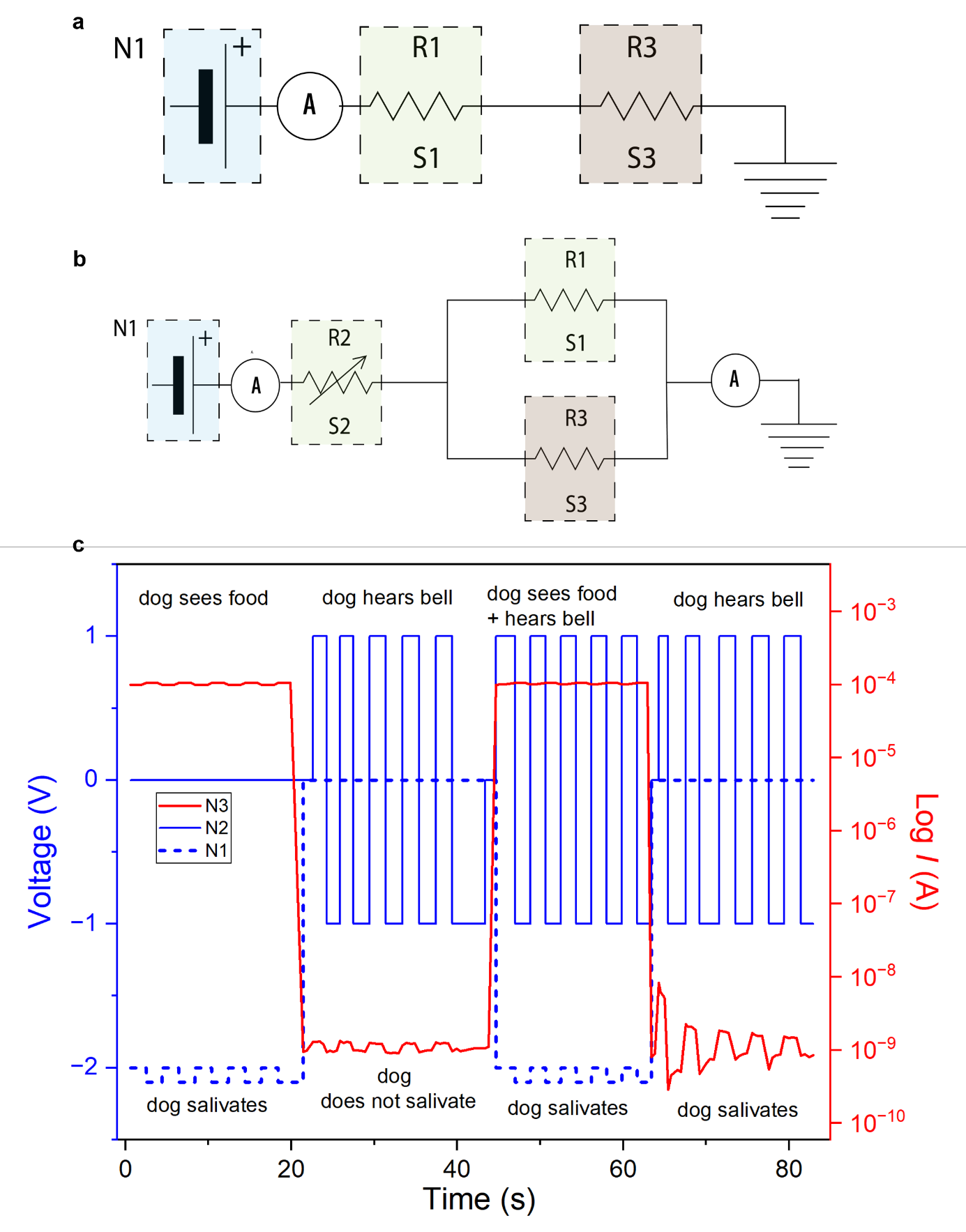
Figure S13.** a) Equivalent circuit for “dog sees food”. b) Equivalent circuit for “Dog hears bell → Dog salivates. c) Full pulse sequence for Pavlov conditioning using two input “neurons” (N1, N2) coupled through a fixed resistor (S1, R1) and the adaptive junction (S2, R2) to the output neuron N3 (R3). The sequence shows consecutively N1-only stimulation (“Dog sees food”), N2-only stimulation (“Dog hears bell”), simultaneous stimulation of N1 and N2 (“Dog sees food and hears bell”, training), and N2-only stimulation after training, where the bell pathway yields an output (“Dog hears bell → Dog salivates”).

# **8. References**

1 C. Gutheil, G. Roß, S. Amirjalayer, B. Mo, A. H. Schäfer, N. L. Doltsinis, B. Braunschweig, F. Glorius, “Tailored Monolayers of N-Heterocyclic Carbenes by Kinetic Control,” *ACS Nano* **18** (2024): 3043–3052, <https://doi.org/10.1021/acsnano.3c08045>.

2 X. Chen, M. Roemer, L. Yuan, W. Du, D. Thompson, E. del Barco, C. A. Nijhuis, “Molecular diodes with rectification ratios exceeding 10^5 driven by electrostatic interactions,” *Nature Nanotechnology* **12** (2017): 797–803, <https://doi.org/10.1038/nnano.2017.110>.

3 C. M. Crudden, J. H. Horton, I. I. Ebralidze, O. V. Zenkina, A. B. McLean, B. Drevniok, Z. She, H. B. Kraatz, N. J. Mosey, T. Seki, et al., “Ultra stable self-assembled monolayers of N-heterocyclic carbenes on gold,” *Nature Chemistry* **6** (2014): 409–414, <https://doi.org/10.1038/nchem.1891>.

4 I. Chiarotto, M. Feroci, A. Inesi, “First direct evidence of N-heterocyclic carbene in BMIm acetate ionic liquids. An electrochemical and chemical study on the role of temperature,” *New Journal of Chemistry* **41** (2017): 7840–7843, <https://doi.org/10.1039/c7nj00779e>.

5 I. Chiarotto, L. Mattiello, F. Pandolfi, D. Rocco, M. Feroci, “NHC in Imidazolium Acetate Ionic Liquids: Actual or Potential Presence?,” *Frontiers in Chemistry* **6** (2018): 355, <https://doi.org/10.3389/fchem.2018.00355>.

6 C. M. Crudden, J. H. Horton, M. R. Narouz, Z. Li, C. A. Smith, K. Munro, C. J. Baddeley, C. R. Larrea, B. Drevniok, B. Thanabalasingam, et al., “Simple direct formation of self-assembled N-heterocyclic carbene monolayers on gold and their application in biosensing,” *Nature Communications* **7** (2016): 12654, <https://doi.org/10.1038/ncomms12654>.

7 A. Bakker, A. Timmer, E. Kolodzeiski, M. Freitag, H. Y. Gao, H. Mönig, S. Amirjalayer, F. Glorius, H. Fuchs, “Elucidating the Binding Modes of N-Heterocyclic Carbenes on a Gold Surface,” *Journal of the American Chemical Society* **140** (2018): 11889–11892, <https://doi.org/10.1021/jacs.8b06180>.

8 Z. Wang, S. Joshi, S. E. Savel’ev, H. Jiang, R. Midya, P. Lin, M. Hu, N. Ge, J. P. Strachan, Z. Li, et al., “Memristors with diffusive dynamics as synaptic emulators for neuromorphic computing,” *Nature Materials* **16** (2017): 101–108, <https://doi.org/10.1038/nmat4756>..

9 D. A. Beattie, A. Arcifa, I. Delcheva, B. A. L. Cerf, S. V. MacWilliams, A. Rossi, M. Krasowska, “pH-Responsive Pickering foams stabilized by silica nanoparticles in combination with trace amount of dodecyl dimethyl carboxyl betaine,” *Colloids and Surfaces A: Physicochemical and Engineering Aspects* **544** (2018): 78–85, <https://doi.org/10.1016/j.colsurfa.2018.02.027>.

10 H. K. Kim, A. S. Hyla, P. Winget, H. Li, C. M. Wyss, A. J. Jordan, F. A. Larrain, J. P. Sadighi, C. Fuentes-Hernandez, B. Kippelen, et al., “Reduction of the Work Function of Gold by N-Heterocyclic Carbenes,” *Chemistry of Materials* **29** (2017): 3403–3411, <https://doi.org/10.1021/acs.chemmater.6b04213>.

11 B. Ball, X. Zhou, R. Liu, “Density functional theory study of vibrational spectra. 8. Assignment of fundamental vibrational modes of 9,10-anthraquinone and 9,10-anthraquinone-d8,” *Spectrochimica Acta Part A: Molecular and Biomolecular Spectroscopy* **52** (1996): 1803–1814, <https://doi.org/10.1016/s0584-8539(96)01769-2>.

12 J. D. Holbrey, W. M. Reichert, I. Tkatchenko, E. Bouajila, O. Walter, I. Tommasi, R. D. Rogers, “1,3-Dimethylimidazolium-2-carboxylate: the unexpected synthesis of an ionic liquid precursor and carbene–CO2 adduct,” *Chemical Communications* (2003): 28–29, <https://doi.org/10.1039/b211519k>.

13 F. Neese, “Software update: The ORCA program system—Version 5.0,” *WIREs Computational Molecular Science* 12 (2022): e1606, <https://doi.org/10.1002/wcms.1606>.

14 M. K. Debe, “Extracting physical structure information from thin organic films with reflection absorption infrared spectroscopy,” *Journal of Applied Physics* **55** (1984): 3354–3366, <https://doi.org/10.1063/1.333374>.

15 M. Wróbel, D. M. Cegiełka, A. Asyuda, K. Kozieł, M. Zharnikov, P. Cyganik, “N-heterocyclic carbenes – The design concept for densely packed and thermally ultra-stable aromatic self-assembled monolayers,” *Nano Today* **53** (2023): 102024, <https://doi.org/10.1016/j.nantod.2023.102024>.

16 H. Kang, J. Jang, G. D. Kong, S. Jung, T. Ohto, H. J. Yoon, “Deposition condition impacts charge tunneling and thermoelectric properties of N-heterocyclic carbene monolayers,” *Journal of Materials Chemistry A* **11** (2023): 16233–16242, <https://doi.org/10.1039/d3ta02443a>.

17 E. Amit, L. Dery, S. Dery, S. Kim, A. Roy, Q. Hu, V. Gutkin, H. Eisenberg, T. Stein, D. Mandler, et al., “Electrochemical deposition of N-heterocyclic carbene monolayers on metal surfaces,” *Nature Communications* **11** (2020): 5714, <https://doi.org/10.1038/s41467-020-19500-7>.

18 A. Abdelmonem, D. Glikman, Y. Gong, B. Braunschweig, H. Saathoff, J. Lützenkirchen, M. H. Fawey, “Surface–bulk photochemical coupling of nonanoic acid and 4-benzoylbenzoic acid: the dual role of the photosensitizer and environmental influences,” *Atmospheric Chemistry and Physics* **25** (2025): 13019–13035, <https://doi.org/10.5194/acp-25-13019-2025>.

19 A. Lagutchev, S. A. Hambir, D. D. Dlott, “Nonresonant Background Suppression in Broadband Vibrational Sum-Frequency Generation Spectroscopy,” *The Journal of Physical Chemistry C* **111** (2007): 13645–13647, <https://doi.org/10.1021/jp075391j>.

20 A. D. Abhayawardhana, T. C. Sutherland, “Heterogeneous proton-coupled electron transfer of a hydroxy-anthraquinone self-assembled monolayer,” *Journal of Electroanalytical Chemistry* **653** (2011): 50–55, <https://doi.org/10.1016/j.jelechem.2011.01.009>.

21 Y. Wang, Q. Zhang, H. P. A. G. Astier, C. Nickle, S. Soni, F. A. Alami, A. Borrini, Z. Zhang, C. Honnigfort, B. Braunschweig, et al., “Dynamic molecular switches with hysteretic negative differential conductance emulating synaptic behaviour,” *Nature Materials* **21** (2022): 1403–1411, <https://doi.org/10.1038/s41563-022-01402-2>.

22 Q. Zhang, Y. Wang, C. Nickle, Z. Zhang, A. Leoncini, D. C. Qi, K. Sotthewes, A. Borrini, H. J. W. Zandvliet, E. del Barco, et al., “Molecular switching by proton-coupled electron transport drives giant negative differential resistance,” *Nature Communications* **15** (2024): 52496, <https://doi.org/10.1038/s41467-024-52496-y>.

23 W. F. Reus, C. A. Nijhuis, J. R. Barber, M. M. Thuo, S. Tricard, G. M. Whitesides, “Statistical Tools for Analyzing Measurements of Charge Transport,” *The Journal of Physical Chemistry C* **116** (2012): 6714–6733, <https://doi.org/10.1021/jp210445y>.

24 Y. Han, C. Nickle, Z. Zhang, H. P. A. G. Astier, T. J. Duffin, D. Qi, Z. Wang, E. del Barco, D. Thompson, C. A. Nijhuis, “Electric-field-driven dual-functional molecular switches in tunnel junctions,” *Nature Materials* **19** (2020): 843–848, <https://doi.org/10.1038/s41563-020-0697-5>.

25 Z. Wang, Z. Li, C. Li, X. Ji, X. Song, X. Yu, L. Wang, W. Hu, “Generic dynamic molecular devices by quantitative non-steady-state proton/water-coupled electron transport kinetics,” *Proceedings of the National Academy of Sciences of the United States of America* **120** (2023): e2304506120, <https://doi.org/10.1073/pnas.2304506120>.

26 Y. Wang, Q. Zhang, C. Nickle, Z. Zhang, A. Leoncini, D.-C. Qi, A. Borrini, Y. Han, E. del Barco, D. Thompson, et al., “Molecular-scale in-operando reconfigurable electronic hardware,” *Nanoscale Horizons* **10** (2025): 349–358, <https://doi.org/10.1039/d4nh00211c>.

27 S. Pecqueur, M. M. Talamo, D. Guérin, P. Blanchard, J. Roncali, D. Vuillaume, F. Alibart, “Neuromorphic Time-Dependent Pattern Classification with Organic Electrochemical Transistor Arrays,” *Advanced Electronic Materials* **4** (2018): 1800166, <https://doi.org/10.1002/aelm.201800166>.

28 Z. Li, X. Yu, “Exploring non-steady-state charge transport dynamics in information processing: insights from reservoir computing,” *Neuromorphic Computing and Engineering* **4** (2024): 024014, <https://doi.org/10.1088/2634-4386/ad54eb>.

29 J. M. Soler, E. Artacho, J. D. Gale, A. García, J. Junquera, P. Ordejón, D. Sánchez-Portal, “The SIESTA method for ab initio order-N materials simulation,” *Journal of Physics: Condensed Matter* **14** (2002): 2745–2750, <https://doi.org/10.1088/0953-8984/14/11/302>.

30 N. Papior, N. Lorente, T. Frederiksen, A. García, M. Brandbyge, “Improvements on non-equilibrium and transport Green function techniques: The next-generation transiesta,” *Computer Physics Communications* **212** (2017): 8–24, <https://doi.org/10.1016/j.cpc.2016.09.022>.

31 J. P. Perdew, K. Burke, M. Ernzerhof, “Generalized Gradient Approximation Made Simple,” *Physical Review Letters* **77** (1996): 3865–3868, <https://doi.org/10.1103/PhysRevLett.77.3865>.

32 S. Grimme, S. Ehrlich, L. Goerigk, “Effect of the damping function in dispersion corrected density functional theory,” *Journal of Computational Chemistry* **32** (2011): 1456–1465, <https://doi.org/10.1002/jcc.21759>.

33 R. C. Chiechi, E. A. Weiss, M. D. Dickey, G. M. Whitesides, “Eutectic Gallium–Indium (EGaIn): A Moldable Liquid Metal for Electrical Characterization of Self-Assembled Monolayers,” *Angewandte Chemie International Edition* **47** (2008): 142–144, <https://doi.org/10.1002/anie.200703642>.

34 D. Bykov, T. Petrenko, R. Izsák, S. Kossmann, U. Becker, E. Valeev, F. Neese, “Efficient implementation of the analytic second derivatives of Hartree–Fock and hybrid DFT energies: a detailed analysis of different approximations,” *Molecular Physics* **113** (2015): 1961–1977, <https://doi.org/10.1080/00268976.2015.1025114>.

35 J. Mao, P. Xu, Z. Zhou, Y. Zhou, Y. Tang, “Electrochemical Evaluation of Self-assembled Monolayers of N-Heterocyclic Carbenes on Gold and DFT Studies,” *Journal of The Electrochemical Society* **168** (2021): 016513, <https://doi.org/10.1149/1945-7111/abdc5d>.
